# Synthesis, Stability, and Kinetics of Hydrogen Sulfide Release of Dithiophosphates

**DOI:** 10.1021/acs.jafc.1c04655

**Published:** 2021-10-25

**Authors:** Eric M. Brown, Nimesh P. R. Ranasinghe Arachchige, Arjun Paudel, Ned B. Bowden

**Affiliations:** Department of Chemistry, University of Iowa, Iowa City, Iowa 52242, United States

**Keywords:** hydrogen sulfide, maize, dithiophosphates, fertilizer, hydrolysis, harvest yield, kinetics

## Abstract

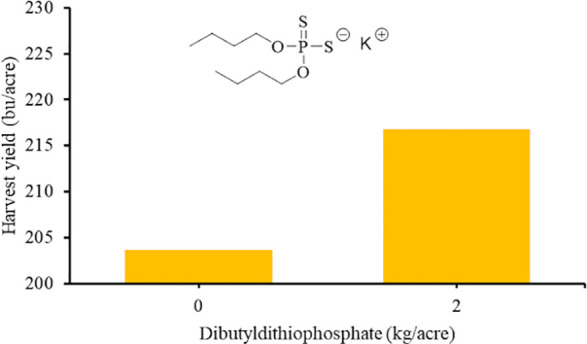

The development of chemicals to slowly release hydrogen sulfide would aid the survival of plants under environmental stressors as well as increase harvest yields. We report a series of dialkyldithiophosphates and disulfidedithiophosphates that slowly degrade to release hydrogen sulfide in the presence of water. Kinetics of the degradation of these chemicals were obtained at 85 °C and room temperature, and it was shown that the identity of the alkyl or sulfide group had a large impact on the rate of hydrolysis, and the rate constant varied by more than 10^4^×. For example, using *tert*-butanol as the nucleophile yielded a dithiophosphate (**8**) that hydrolyzed 13,750× faster than the dithiophosphate synthesized from *n*-butanol (**1**), indicating that the rate of hydrolysis is structure-dependent. The rates of hydrolysis at 85 °C varied from a low value of 6.9 × 10^–4^ h^–1^ to a high value of 14.1 h^–1^. Hydrogen sulfide release in water was also quantified using a hydrogen sulfide-sensitive electrode. Corn was grown on an industrial scale and dosed with dibutyldithiophosphate to show that these dithiophosphates have potential applications in agriculture. At a loading of 2 kg per acre, a 6.4% increase in the harvest yield of corn was observed.

## Introduction

Gasotransmitters are key chemicals that are synthesized by enzymes and used for intra and intercellular signaling, and hydrogen sulfide (H_2_S) is the newest gasotransmitter reported in plants^1−3^ and humans.^4−6^ Much of the research with H_2_S in living systems has been completed with human cells, and some important concepts have emerged. In humans, it is synthesized by several enzymes, including cystathionine-β-synthase,^4,5^ cystathionine-γ-lyase,^4,5^ and 3-mercaptopyruvate sulfurtransferase,^6^ and it has been shown to affect dozens of enzymatic pathways in eukaryotic cells. H_2_S affects enzymes by cleaving disulfides,^7^ converting thiols or disulfides into persulfides,^7,8^ and scavenging reactive oxygen species.^4,9,10^ Enzymes that synthesize H_2_S have been reported in plants, and recent work has shown that low doses of H_2_S can increase the growth of a wide variety of most important crops grown in the United States including corn (*Zea mays*),^11−13^ peas (*Pisum sativum*),^14,15^ lettuce (*Lactuca sativa*),^14,16,17^ soybeans (*Glycine max*),^18,19^ and more.^2,14,20−22^ In two papers in 1978 and 1979, Thompson et al. reported that continuous levels of 300 and 3000 ppm of H_2_S in the atmosphere of a greenhouse reduced the growth of lettuce, sugar beets (*Beta vulgaris* subsp. *vulgaris* var. *altissima*), and alfalfa (*Medicago sativa*), but 30 to 100 ppb of continuous H_2_S in a greenhouse promoted their growth as shown by the increase in the harvest weight of lettuce up to 61% and the increase in the harvest weight of sugar beets by up to 54%.^16,17^ This research was largely ignored, and H_2_S was believed to be toxic at all loadings in plants. It was not until 2005 when the discovery of an H_2_S-producing enzyme in plants^1^ caused scientists to more fully investigate its effect on plants.

Recent research has shown that low doses of H_2_S aid the survival of plants to environmental stressors and increase their rates of germination. Plants exposed to H_2_S have been shown to better survive heat, drought, salt, heavy metals, and other stressors better than plants not exposed to H_2_S.^12,13,15,18−22^ These differences in survival of plants are large, and papers often report a doubling of survival of plants exposed to H_2_S compared to plants not exposed to H_2_S. The amount of H_2_S needed to have these beneficial effects is not well understood because of the difficulties in delivering a controlled amount of a low-boiling point gas, but the trend has been observed for numerous different types of plants and with different mechanisms to expose them to H_2_S. The mechanism of how H_2_S affects plants is an active area of research, but it is clear that H_2_S is emerging as a key chemical in plant growth.

**Figure 1:**
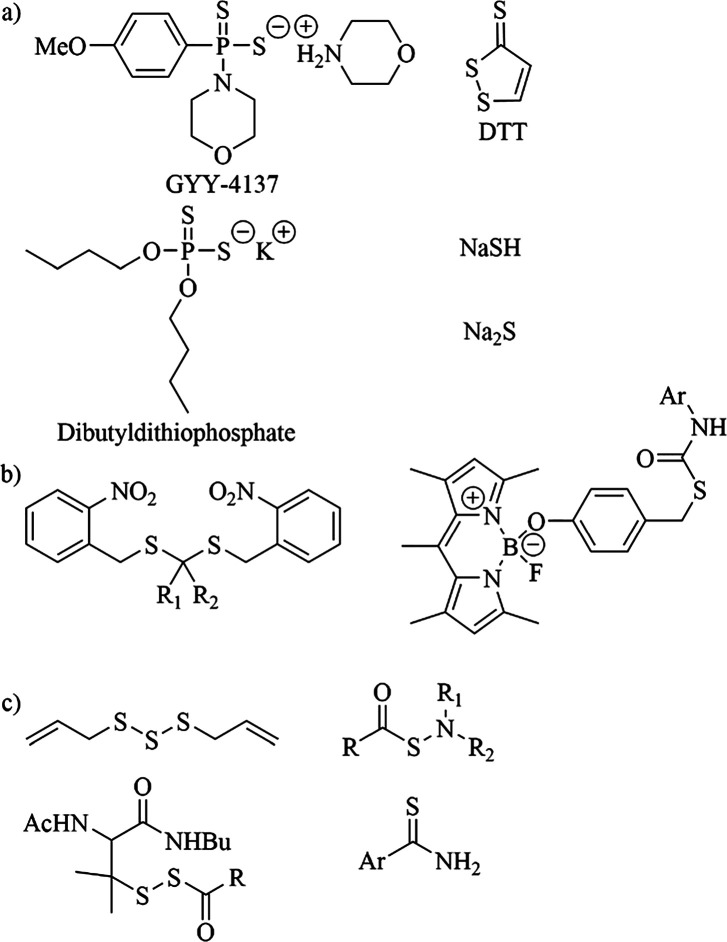
Chemicals that release H_2_S in response to (a) hydrolysis, (b) light, and (c) presence of thiols. DTT have also been shown to release H_2_S in the presence of thiols.

A major challenge in developing agricultural applications of H_2_S is that it is a low-boiling point gas (boiling point = −60 °C) and highly toxic at ppm concentrations.^23^ To address this challenge, chemicals that release H_2_S in response to stimulus such as hydrolysis,^11,24−27^ light,^28,29^ or the presence of thiols^30−32^ have been developed (Figure 1). Chemicals that slowly release H_2_S by hydrolysis are typically used to deliver low doses of H_2_S over extended periods of time from days to weeks to months. The long release of H_2_S may be beneficial in agricultural applications to increase the survival and harvest yields of crops. In this article, we report on a series of dithiophosphates that are synthesized in one step and release H_2_S by hydrolysis at rates that span over four orders of magnitude. We show how the structure and chemical composition of dithiophosphates affect their rates of hydrolysis to release H_2_S. In addition, we report how a dithiophosphate improves the harvest yield of corn grown in field trials. This paper provides a method to design dithiophosphates to release H_2_S at time periods and at amounts that can be tuned to optimize the positive effects of H_2_S on a wide variety of plants.

Two of the commonly used chemicals to slowly deliver constant doses of H_2_S are GYY-4137^14,24,33^ and 1,2-dithiole-3-thiones (DTT)^25,34,35^ (Figure 1). Both of these chemicals slowly release H_2_S through hydrolysis or when exposed to thiols, and they yield constant, low levels of H_2_S that are desired for plants. A sustained increase in sulfide concentration over 3 h was observed in rat plasma upon addition of GYY-4137 in vivo, showing that a constant low-level dosage of H_2_S is also released in cells.^24^ In prior work by us and others, it was shown that <3% of GYY-4137 hydrolyzed to release H_2_S in water at room temperature after 35 days and that 50% hydrolyzed in CDCl_3_ using residual water at room temperature after 13 days.^14,33^ This showed that the environment of GYY-4137 can strongly impact its rate of hydrolysis. Chemicals that release H_2_S by reaction with thiols or with light have been reported, but in this paper, we emphasize the release of H_2_S by hydrolysis with water because of the slow, steady release of H_2_S over weeks to months through this mechanism. Although these chemicals are widely used, there is a need to synthesize chemicals that hydrolyze in water to release H_2_S over a wider range of rates to allow the rate of release of H_2_S to be tuned to have the desired effect on plants.

**Figure 2:**
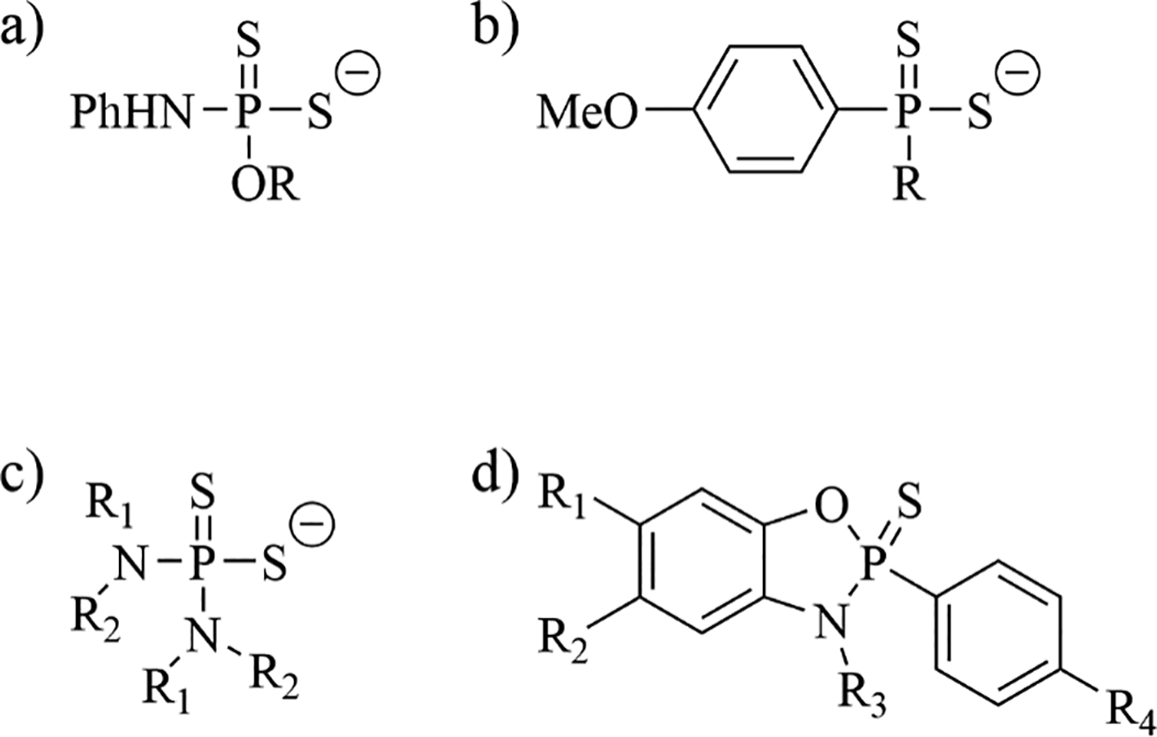
General structures of phosphordithioates synthesized (a) from a four-step procedure developed by Park et al. and (b) from Lawesson’s reagent are shown. (c) Phosphordithioates with amines bonded to the phosphorous release H_2_S significantly faster than GYY-4137, but they are unstable in air. The phosphordithioates shown in (d) release H_2_S faster than GYY-4137 and are stable.

Two recent papers report the synthesis of a series of chemicals related to GYY-4137 that resulted in chemicals with different rates of hydrolysis. Park et al. developed a four-step synthesis of five chemicals with a phosphordithioate core (Figure 2a). The hydrolysis of these chemicals was measured for 3 h, and they released H_2_S at similar rates to GYY-4137.^26^ This work demonstrated that the structure around the phosphorus needed to be more widely varied to have a wider rate of hydrolysis. Feng et al. reported 27 different chemicals with a phosphordithioate core that were synthesized from starting materials such as Lawesson’s reagent (Figure 2b).^27^ The amounts of H_2_S released after 4 days from these chemicals were measured in 10% H_2_O/90% CH_3_CN (v/v) using a fluorescent dye and compared to GYY-4137. All of the 14 chemicals synthesized from Lawesson’s or Belleau’s reagents yielded similar rates of release of H_2_S as measured for GYY-4137 (Figure 2b). To achieve higher rates of release of H_2_S, four chemicals with the general structure shown in Figure 2c were synthesized where amines were bonded to the phosphorous. These chemicals were hygroscopic and degraded in air rapidly, and two of them released >48% of the sulfur as H_2_S over two days. Because of the low stability of the amine-based chemicals and their rapid release of H_2_S, nine chemicals with structures shown in Figure 2d were synthesized to further explore how to vary the rate of release of H_2_S. These chemicals were stable and showed faster rates for the release of H_2_S compared to GYY-4137. This report demonstrated that the groups attached to the phosphordithioate core needed to be varied to have a wide range of H_2_S release. It also demonstrated that measuring the amount or rate of release of H_2_S in water can be challenging because of the solubility of the chemicals and the limited methods to quantify how much H_2_S has been released. The use of fluorescent dyes to measure the amount of H_2_S released resulted in a limited number of data points, and no rate constants were reported. The results are highly interesting and suggest that a systematic manipulation of the phosphordithioate core may yield new discoveries.

We have interest in investigating how chemicals that release H_2_S affect the growth, survival, and harvest yields of plants. In recent work, we showed that milligram loadings of GYY-4137 and dibutyldithiophosphate increased the harvest yields of radishes (*Raphanus sativus*), peas, and lettuce and increased the growth of corn plants.^11,14^ The mechanism for how H_2_S has an important effect on plants is poorly understood, although it is likely that it acts on plant enzymes similar to how it has been shown to act on enzymes in human cells.^4,7−10^ The application of exogenous H_2_S in agriculture is a new and potentially useful target to promote the survival of plants in a changing climate and to improve the harvest yields of many crops. To fully investigate these opportunities in agriculture, a range of chemicals that slowly release H_2_S over part or all of the lifecycle of a plant are needed. These chemicals should release H_2_S at low concentrations to promote the growth of plants but not so high as to be detectable above ground and cause health problems for farmers or scientists who study these chemicals. Therefore, it is important to understand their rates of hydrolysis.

In this paper, we describe the synthesis and release of H_2_S from a series of dithiophosphates to investigate how the structure and identity of the atoms bonded to the phosphorous affect their rates of hydrolysis and release of H_2_S. Dithiophosphates have potential to be used in commercial applications because they react with water to release H_2_S, phosphate (a commonly used fertilizer), and the chemicals used in their synthesis. By selecting natural chemicals to synthesize dithiophosphates, the degradation products of hydrolysis will be safe for the environment and not contribute to pollution. Prior work demonstrated that the rate of hydrolysis of chemicals with the phosphodithioate core was sensitive to their structure, but it was not fully understood how the structure around phosphorous affected their rates of hydrolysis.^27^ We chose to investigate how dithiophosphates synthesized from a wide range of different functional groups including primary, secondary, and tertiary alcohols; thiols; diols; phenol; thiophenol; and mercaptoethanol affected their rates of hydrolysis in water.

## Experimental Procedures

### Materials and Methods

All chemicals were obtained from Sigma-Aldrich at their highest purity and used as received. Nuclear magnetic resonance (NMR) spectra were obtained using a BrukerAvance-300 at 300 MHz, a Bruker DRX-400 at 400 MHz, and a Bruker DPX-500 at 500 MHz. An amperometric H_2_S microsensor for real-time H_2_S monitoring was purchased from Analysenmesstechnik GmBH.

### Synthesis of Dithiophosphates

The synthesis of the dithiophosphates followed the same general procedure, and the synthesis of each dithiophosphate is described in detail in the Supporting Information. The synthesis of dithiophosphate **3** is described here as one example. 1,2-Propanediol (0.45 g and 6 mmol) was added slowly over 2 min to a mixture of P_2_S_5_ (0.65 g and 3 mmol) and toluene (20 mL). The contents were stirred at 90 °C for 12 h. To obtain crude compound **3**, the contents were cooled in an ice bath, and 8 mL of 0.75 M potassium hydroxide was added slowly over 2 min. The crude product was dried under reduced pressure and purified by flash chromatography using a solvent system of 20% methanol in ethyl acetate as an eluent to yield a white solid (65% yield). ^1^H NMR (300 MHz, CD_3_OD) δ = 4.80–4.85 (m, 1H), 4.54–4.59 (m, 1H), 3.75–3.82 (q, 1H), 1.35–1.37 (d, 3H), ^31^P NMR (300 MHz, CD_3_OD) δ = 129.33. ESI-MS *m*/*z* [M+] calculated: 168.9552, found: 168.9541.

### Hydrolysis of Chemical 2 at Room Temperature in 90% H_2_O/D_2_O Measured by ^31^P NMR Spectroscopy

The hydrolysis of the dithiophosphates was performed using the same general procedure. The procedure for dithiophosphate **2** is described here, and the procedures for the other dithiophosphates are described in the Supporting Information. Dithiophosphate **2** (81.2 mg, 0.32 mmol) was dissolved in 1.5 mL of 90% H_2_O/D_2_O, yielding a 0.21 M solution. The ^31^P NMR spectra (300 MHz, 90% H_2_O/D_2_O) were collected at day 0 and day 30.

### ^31^P NMR spectroscopy of Hydrolysis of Dithiophosphates at 85 °C

A similar procedure was followed to determine the rate of hydrolysis of the dithiophosphates. The procedure for dithiophosphate **3** is described here, and the remaining procedures are found in the Supporting Information. Dithiophosphate **3** (10.4 mg and 0.05 mmol) was dissolved in 1 mL 90% H_2_O/D_2_O, yielding a 50 mM solution. The solution was added to an NMR tube and placed in an 85 °C oil bath. ^31^P NMR spectra (300 MHz) were taken periodically to measure the degradation.

### H_2_S Release from Dithiophosphates Measured by an H_2_S Electrode

To a glass jar, 70 mL of phosphate-buffered H_2_O (pH 6.7) was added. A baseline of the concentration of H_2_S was measured for half an hour to confirm that it was zero. Next, dithiophosphates were added to the buffered water to yield different concentrations. The jar was capped with a rubber stopper that had a hole cut into it for the H_2_S and pH electrodes. The measurements of the concentration of sulfide and pH were logged into a spreadsheet every 2 s.

### Field Trial of Corn with Dibutyldithiophosphates

The field trials were completed on a farm near Ames, Iowa in the summer of 2020. Prior to planting the corn, the fields were fertilized with 180 lb nitrogen per acre as 32% urea ammonium nitrate, 60 lb per acre of P_2_O_5_, 80 lb per acre of K_2_O, and 20 lb per acre of sulfur. The corn seeds were a 110 day maturity hybrid with an herbicide and insect protection gene package. The corn seeds were planted with a commercial liquid starter fertilizer (2-40-28) delivered in furrow with the seeds. Prior to planting the seeds, dibutyldithiophosphate was added to the starter fertilizer to achieve a concentration of 0, 0.5, 1.0, or 2.0 kg per acre of dibutyldithiophosphate. The dithiophosphate was dissolved in the starter fertilizer hours before it was applied, and the starter fertilizer was added as a constant stream in a row where the seeds were planted. Five gallons of starter fertilizer and dithiophosphate were used per acre. The corn seeds were planted at a rate of 35,000 seed per acre. The corn seeds were planted on April 30, 2020 and harvested on September 26, 2020. The harvest weight was determined for each plot and corrected for slight differences in moisture content. The protein level, oil level, starch level, and density of the corn grown with 0 and 2 kg per acre of dibutyldithiophosphate were measured and found to be the same for both sets.

## Results and Discussion

### Synthesis of Dialkoxydithiophosphates

**Figure 3:**
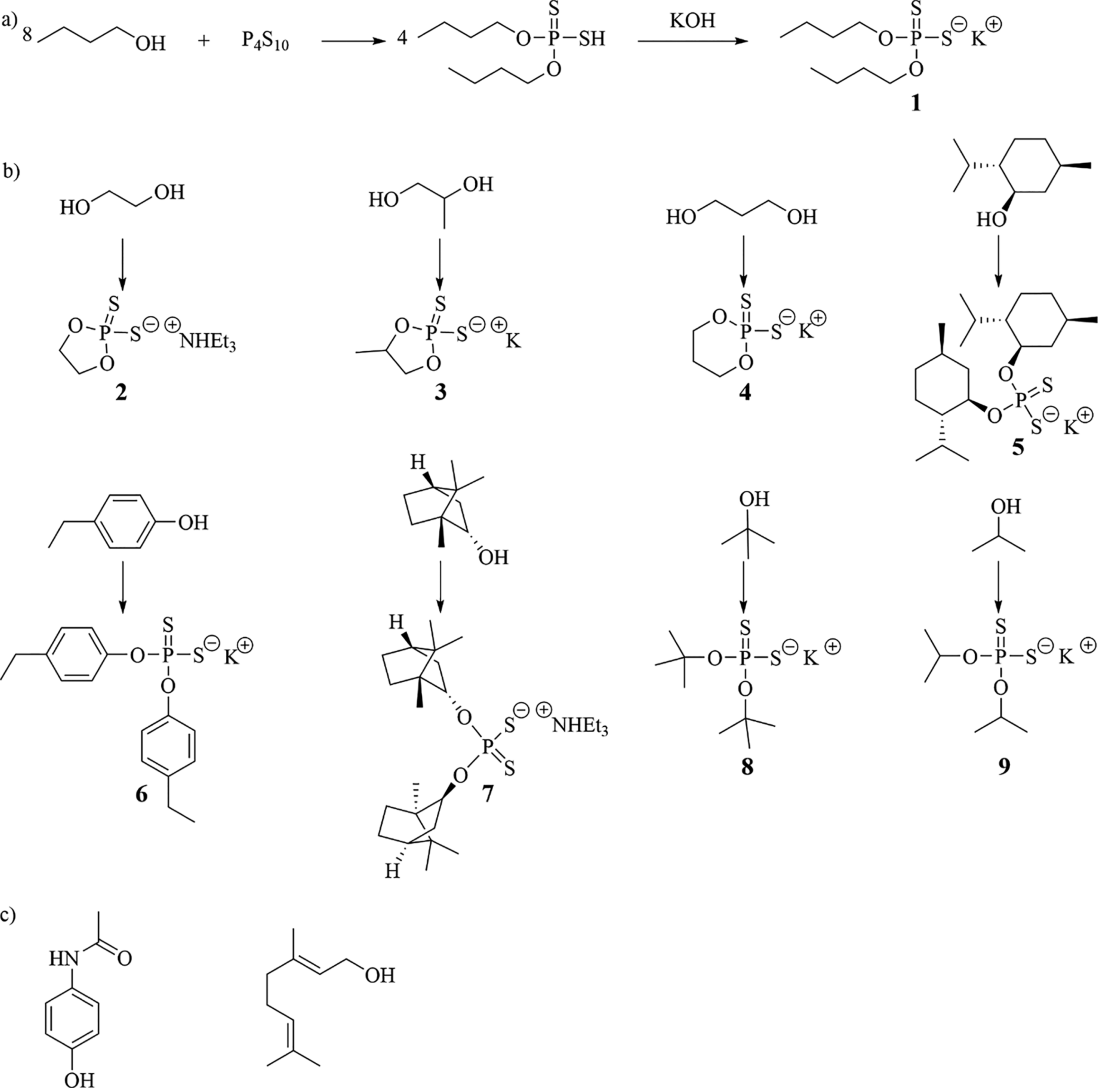
(a) General reaction scheme for the synthesis of dialkoxydithiophosphates. (b) Starting alcohols used to synthesize dialkoxydithiophosphates are shown. (c) Reactions with P_4_S_10_ and acetaminophen (left) and geraniol (right) were unsuccessful.

In a recent publication, we reported the synthesis of dithiophosphates synthesized from the C2 to C12 fatty alcohols and P_4_S_10_ at an elevated temperature of 85 °C for up to 16 h (Figure 3a).^11^ The acids slowly decomposed at room temperature, so the salts were isolated and shown to be stable at room temperature under ambient conditions for months. These reaction conditions were used as the starting point for the synthesis of the dialkoxydithiophosphates reported here, and the syntheses were successful to yield the dialkoxydithiophosphates with yields from 66 to 93% (Figure 3b). These reactions included the synthesis of dialkoxydithiophosphates using the naturally occurring terpenes *L*-menthol and (−)-borneol (**5** and **7**). The potassium salt was isolated for each, except **2** and **7** where the triethylamine salts were isolated because of difficult purification of **2** and poor solubility of **7** as the potassium salt.

The reactions to synthesize dialkoxydithiophosphates **2** and **8** from *t*-butanol and ethylene glycol were completed at 45 °C. The dithiophosphate synthesized from *t*-butanol was sensitive to elevated temperatures, and undesired side products were formed at 85 °C, so a lower temperature was successfully investigated. The reaction with ethylene glycol at 85 °C gave numerous side products making purification difficult.

Attempts to synthesize the dithiophosphates from acetaminophen and geraniol led to numerous products in the crude ^1^H NMR spectra (Figure 3c). P_4_S_10_ is known to convert amides to thioamides, and this reaction occurred when acetaminophen was used.^36^ The product formed from the reaction of geraniol and P_4_S_10_ may have been unstable because of the presence of an allyl group.

### Synthesis of Disulfidedithiophosphates

**Figure 4:**
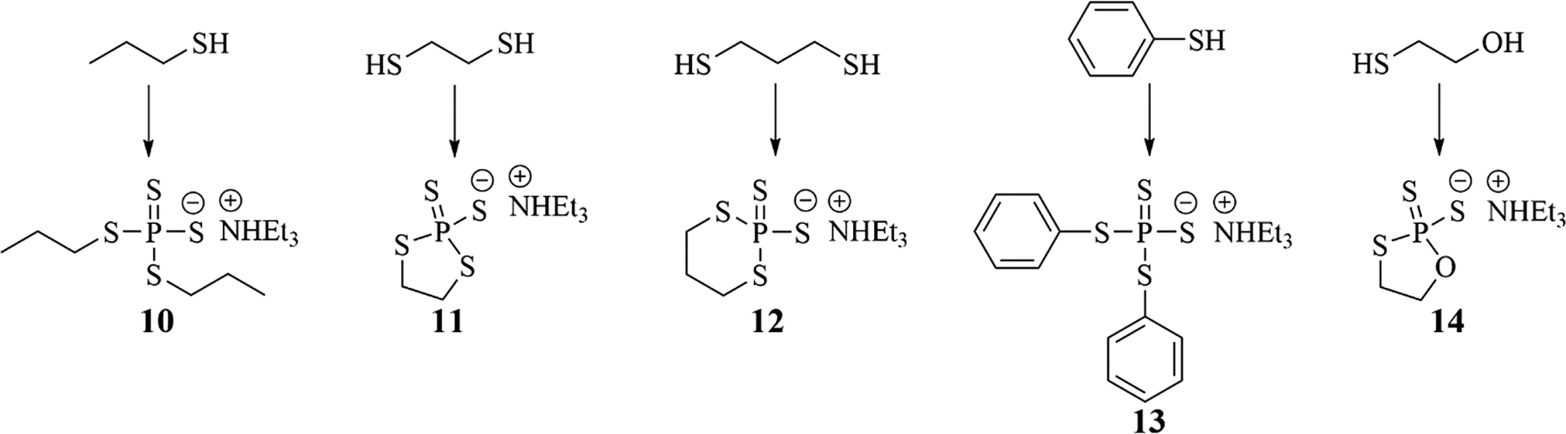
Synthesis of disulfidedithiophosphates from different thiols is shown.

Disulfidedithiophosphates were also pursued because of the natural occurrence of thiols in plants. Thiols were more reactive than fatty alcohols with P_4_S_10_ so the reactions shown in Figure 4 were completed at 40 °C. These reactions proceeded to high yields, and the products were obtained as triethylamine salts because the potassium salts were insoluble in most solvents including water.

### Synthesis of Diaminodithiophosphates

**Figure 5:**
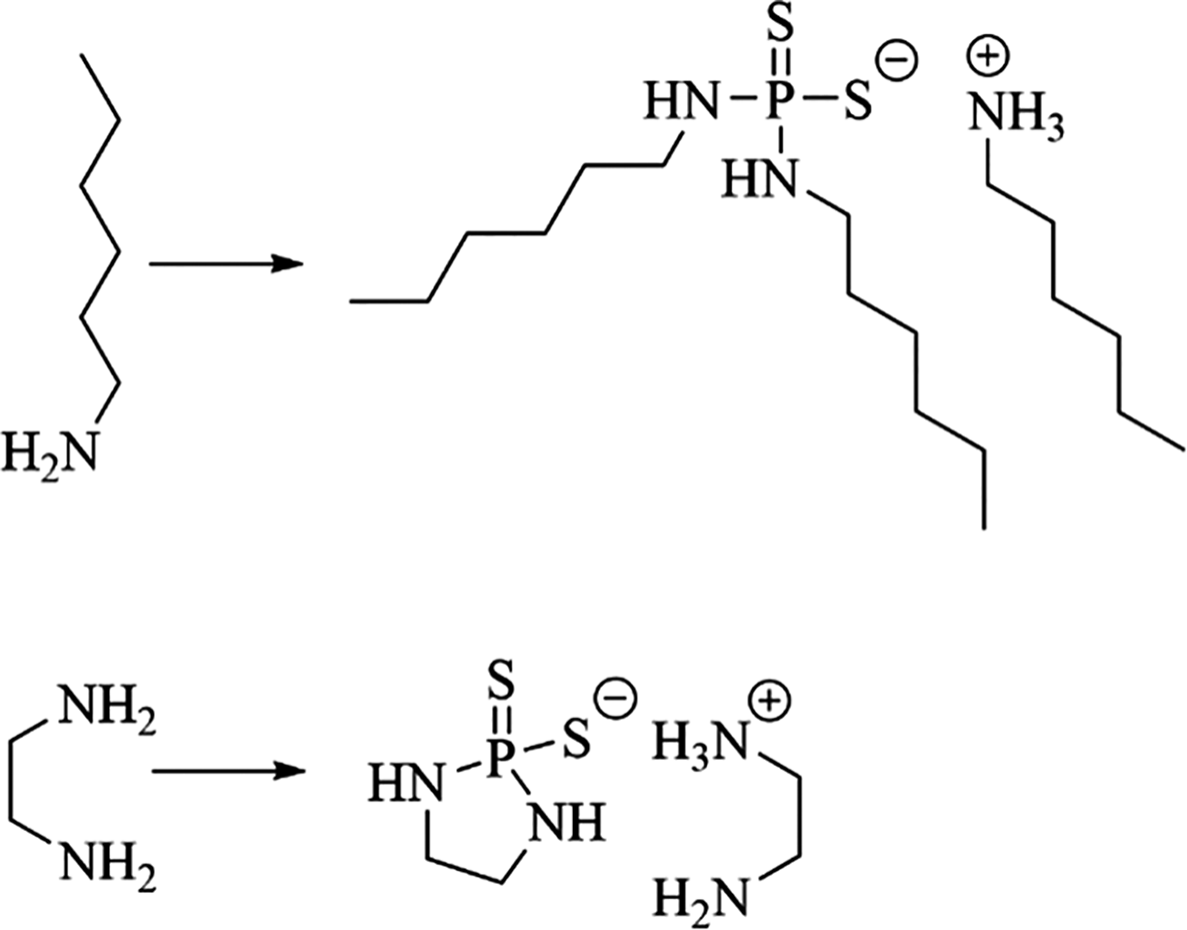
Synthesis of dihexylaminodithiophosphate (top) and ethylenediaminodithiophosphate (bottom) was attempted but they could not be purified because of rapid degradation in air.

Prior work suggested that diaminodithiophosphates were not stable and released H_2_S very quickly, and similar properties were observed.^27^ The reactions of hexylamine and ethylene diamine with P_4_S_10_ were completed at room temperature, but neither product could be isolated in high purity (Figure 5). The solid products that were initially isolated decomposed upon exposure to atmospheric conditions to yield thick liquids with the notable release of H_2_S. These chemicals possessed strong odors of H_2_S that required them to be housed in sealed containers. In contrast, the dialkoxydithiophosphates and disulfidedithiophosphate salts were stable for months and possessed little to no odor of H_2_S. Because of the intense odor of H_2_S from diaminodithiophosphates and their rapid reaction with atmospheric water to release H_2_S, these chemicals are unlikely to find applications as fertilizers in agriculture and were not investigated further.

### Hydrolysis of Dithiophosphates in D_2_O/H_2_O

The hydrolysis of dithiophosphates was tracked by ^31^P NMR spectroscopy to investigate their stabilities at room temperature (23–25 °C) when dissolved in 90% H_2_O/D_2_O for 30 days (Table 1). The chemicals were added to NMR tubes and dissolved in 90% H_2_O/D_2_O, and the ^31^P NMR spectra were measured on days 0 and 30. In our prior work, less than 3% of the dithiophosphates synthesized from fatty alcohols hydrolyzed after 35 days, and the results shown in Table 1 were consistent with that observation.^11^ Of the 13 chemicals investigated, 8 of the dithiophosphates showed less than 3% hydrolysis, and 3 others were not soluble in water and not investigated. The potassium and triethylamine salts of **5**, **7**, and **13** were synthesized in attempts to improve their solubilities, but these salts were insoluble in water.

**Table 1:** Degradation of Dithiophosphates Was Followed by ^31^P NMR Spectroscopy for 30 Days at Room Temperature in 90% H_2_O/D_2_O

chemical	degradation (%)
**1**^a^	<3
**2**	<3
**3**	<3
**4**	<3
**5**	IS^b^
**6**	<3
**7**	IS
**8**	100
**9**	<3
**10**	<3
**11**	<3
**12**	<3
**13**^c^	<3
**14**	100

a The hydrolysis of **1** was reported in a previous publication.

b IS, insoluble.

c A 3:1 DMSO/water mixture was used because of the compound being insoluble in water.

Surprisingly, **8** and **14** were completely hydrolyzed in 90% H_2_O/D_2_O after 30 days. We further investigated their hydrolysis, and **14** was 60% hydrolyzed after 4 days and completely hydrolyzed on day 13. This result was in contrast to **2**, **3**, and **11** that also had five-membered rings with either oxygen or sulfur. Dithiophosphate **8** hydrolyzed slower than **14**, and at days 12 and 20, it was 62 and 88% degraded. The hydrolysis of **8** was much faster than the dithiophosphates synthesized from primary or secondary alcohols.

### Kinetics of Hydrolysis of Dithiophosphates

The hydrolysis of the dithiophosphates was investigated in 90% H_2_O/D_2_O at 85 °C by ^31^P NMR spectroscopy. This temperature was chosen to accelerate the hydrolysis that was very slow at room temperature. The dithiophosphates were dissolved at known concentrations, and the NMR tubes were placed in an oil bath at 85 °C. The NMR tubes were periodically removed from the oil bath, the ^31^P NMR spectra were collected, and the NMR tubes were placed back in the 85 °C oil bath.

**Figure 6:**
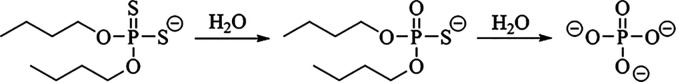
Mechanism of hydrolysis of dibutyldithiophosphate is shown.

The hydrolysis of the dithiophosphates followed pseudo-first-order reaction rates. The fit of the hydrolysis of these chemicals to first- and zero-order reactions is shown in Figures S40–S53 to demonstrate that the better fits occurred for the first-order kinetics. In our prior work, we showed that the hydrolysis of dibutyldithiophosphate followed the mechanism shown in Figure 6.^11^ The first step of the hydrolysis was release of H_2_S, and the oxo intermediate was observed in the ^31^P NMR spectra. No other intermediates were observed during this reaction for dibutyldithiophosphate. In the hydrolysis at 85 °C for the dithiophosphates reported here, low concentrations of multiple intermediates were only observed for dithiophosphates **14** and **4**. The remaining chemicals displayed one or no intermediates. All of the chemicals degraded to release phosphoric acid. The rate constant of the first step in the reaction, half lives (*t*_1/2_) of the first step, and time for the dithiophosphates to completely hydrolyze to yield phosphoric acid are shown in Table 2.

**Table 2:** Rate Constant and Half-Life for the First Step in the Hydrolysis^a^

chemical	rate of the first step of hydrolysis (85 °C)	*t*_1/2_ (days)	time to complete hydrolysis^b^
1	9.6 × 10^–4^ h^–1^	30	180 days
2	3.4 × 10^–3^ h^–1^	8.5	49 days
3	7.1 × 10^–3^ h^–1^	4.1	23 days
4	6.9 × 10^–4^ h^–1^	42	200 days
6	7.4 × 10^–3^ h^–1^	3.9	10 days
8	13.2 h^–1^	0.0021	15 min
9	1.7 × 10^–3^ h^–1^	17	85 days
10	2.2 × 10^–1^ h^–1^	0.13	0.65 days
11	3.4 × 10^–1^ h^–1^	0.085	0.44 days
12	1.9 × 10^–2^ h^–1^	1.5	9 days
13^c^	2.2 × 10^–2^ h^–1^	1.3	7 days
14	14.1 h^–1^	0.0020	15 min
8 (room temperature)	5.1 × 10^–3^ h^–1^	5.7	30 days
14 (room temperature)	1.1 × 10^–2^ h^–1^	2.9	12 days

a In addition, time to complete hydrolysis of the dithiophosphates to phosphoric acid is shown.

b The hydrolysis was labeled complete when the corresponding dithiophosphate was no longer observed by ^31^P NMR.

c A 3:1 DMSO/water mixture was used.

The hydrolysis of **8** and **14** was rapid and completed within 90 min at 85 °C so their rates of hydrolysis were measured at room temperature and are shown in Table 2. Their rates of hydrolysis were 2600× for **8** and 1300× for **14** slower at room temperature than at 85 °C, but both chemicals were completely hydrolyzed within 30 days at room temperature.

The results in Table 2 show several important findings about how the structure of the dithiophosphates affects their rates of hydrolysis. Dialkoxydithiophosphates synthesized with primary, secondary, and tertiary alcohols have relative rates of 1.00: 1.78: 13,800. The large difference in rates of hydrolysis for dithiophosphates synthesized from tertiary to secondary and primary alcohols was unexpected.

Comparing the rates of hydrolysis of dialkoxydithiophosphates to disulfidedithiophosphates shows that the disulfidedithiophosphates hydrolyze much faster. Dithiophosphate **10**, synthesized from a primary thiol, had a rate of hydrolysis 230× faster than **1** which was synthesized from a primary alcohol. The difference between the rates of hydrolysis was much smaller when the dithiophosphate synthesized using ethylphenol (**6**) was compared to the dithiophosphate synthesized from thiophenol (**13**). The hydrolysis of **13** was only 3.0× faster than that of **6**. The hydrolysis of **6** was 8.0× faster than the hydrolysis of **1**, but when the oxygen was replaced with sulfur the hydrolysis of the thiophenol dithiophosphate (**13**) was 10× faster than the hydrolysis of the primary thiol dithiophosphate (**10**). To confirm that the increased rate of hydrolysis was not due to the change in the counter ion, the triethylamine salt of **8** was synthesized, and a similar rate of hydrolysis to the potassium salt was observed (Figure S61).

Prior work showed that the phosphates synthesized from diols such as ethylene glycol hydrolyze up to 10^5^ faster than similar phosphates synthesized from primary alcohols.^37,38^ Although the heterosubstituted five-membered ring in phosphate synthesized from ethylene glycol (**2**) was strained, it was shown that the difference for rates of hydrolysis was related to differences in solvation. This trend was not observed with dithiophosphates synthesized from diols or disulfides. The dithiophosphate synthesized from *n*-butanol (**1**) hydrolyzed 3.5× slower than five-membered ring dithiophosphate **2**, 7.4× slower than the five-membered ring dithiophosphate **3**, and 1.4× faster than the six-membered ring dithiophosphate **4**. Interestingly, the rates of hydrolysis of similarly structured disulfidedithiophosphates with five- (**11**) and six- (**12**) membered rings containing sulfur atoms were within 2× of the rate of hydrolysis of **10**.

It is possible that the rates of hydrolysis follow a different mechanism from that shown in Figure 6. For instance, the first hydrolysis may be the release of an alcohol or thiol from the phosphate rather than the loss of H_2_S. Despite the lack of clarity about the first step in the hydrolysis, all of the dithiophosphates release H_2_S in water. In a later section, the release of H_2_S from the dithiophosphates was measured using an H_2_S electrode, and each of them released H_2_S immediately upon immersion in water.

### Free Energy Values of the Transition State of Select Dithiophosphates

To better understand why the rates of hydrolysis were rapid for **8** and **14** at room temperature and 85 °C, the rate constants were measured for **8**, **10**, **11**, and **14** at a variety of temperatures to extract the values for Δ*H*^‡^ and Δ*S*^‡^. The hydrolysis of phosphates has been well studied in the literature because of their importance in RNA, DNA, and more.^39−42^ Most mechanisms of hydrolysis proceed by a two-step SN_2_P mechanism with the incoming nucleophile attacking the phosphorous followed by an elimination step.^40−42^ Computational studies of the mechanism of hydrolysis mostly confirm a two-step mechanism,^43−45^ but in some studies, only one transition state was observed when a sulfur replaced one of the oxygens in the chemical being investigated.^46,47^ Furthermore, some hydrolysis mechanisms follow a SN_1_P mechanism where the phosphate loses an alcohol before the nucleophile attacks the phosphorous.^48,49^ Prior work of the hydrolysis of phosphates reveals a rich and complex set of mechanisms that are dependent on the pH, ionic concentration, and structure of the phosphates.^39−45^

The rate constants for the hydrolysis of **8**, **10**, **11**, and **14** were measured at temperatures from 25 to 85 °C as described in the Supporting Information. These chemicals were selected based on their relatively rapid rates of hydrolysis that would allow these rates to be measured in a wide range of temperatures and to provide structurally similar chemicals to **8** and **14** to compare their rates of hydrolysis. The values for Δ*H*^‡^ and Δ*S*^‡^ were calculated and are reported in Table 3.

**Table 3:** Enthalpy, Entropy, and Free Energy Values of the Transition State of Compounds **8**, **10**, **11**, and **14**

chemical	Δ*H*^‡^ (kJ/mol)	Δ*S*^‡^ (J/mol·K)	–*T*Δ*S*^‡^ (25 °C) (kJ/mol)	Δ*G*^‡^ (25 °C) (kJ/mol)	Δ*G*^‡^ (85 °C) (kJ/mol)	ΔΔ*G*^‡^ (kJ/mol)
**8**	116.5	36.3	–10.8	105.7	103.5	2.2
**10**	77.6	–109.6	32.7	110.2	116.8	–6.6
**11**	137.1	58.4	–17.4	119.7	116.2	3.5
**14**	104.3	2.46	–0.733	103.5	103.4	0.1

Although Δ*H*^‡^ ranged from a low value of 77.6 to a high value of 137.1 kJ mol^–1^, the most interesting values are those for Δ*S*^‡^. The Δ*S*^‡^ of **10** was strongly negative which is consistent with a SN_2_P mechanism^43−45^ and similar to numerous other values for Δ*S*^‡^ found for the hydrolysis of dithiophosphates.^50,51^ In contrast, the values for Δ*S*^‡^ were positive for **8**, **11**, and **14** although the value for **14** was close to zero. The interpretation for the positive values of Δ*S*^‡^ was unclear because of the several mechanisms of hydrolysis of phosphates that have been reported. The positive Δ*S*^‡^ values may be due to the hydrolysis following a SN_1_P mechanism, following a SN_2_P mechanism and possessing the elimination of a group from the phosphate as the rate-determining step, or by following a mechanism other than the SN_1_P or SN_2_P mechanisms.

The data in Table 3 allow the rate of hydrolysis of four dithiophosphates to be calculated at any reasonable temperature encountered in an agricultural setting. This result is important because it allows the release of H_2_S to be correlated to their effect on plants.

### H_2_S Release from Dithiophosphates Measured Using an H_2_S Electrode

**Figure 7:**
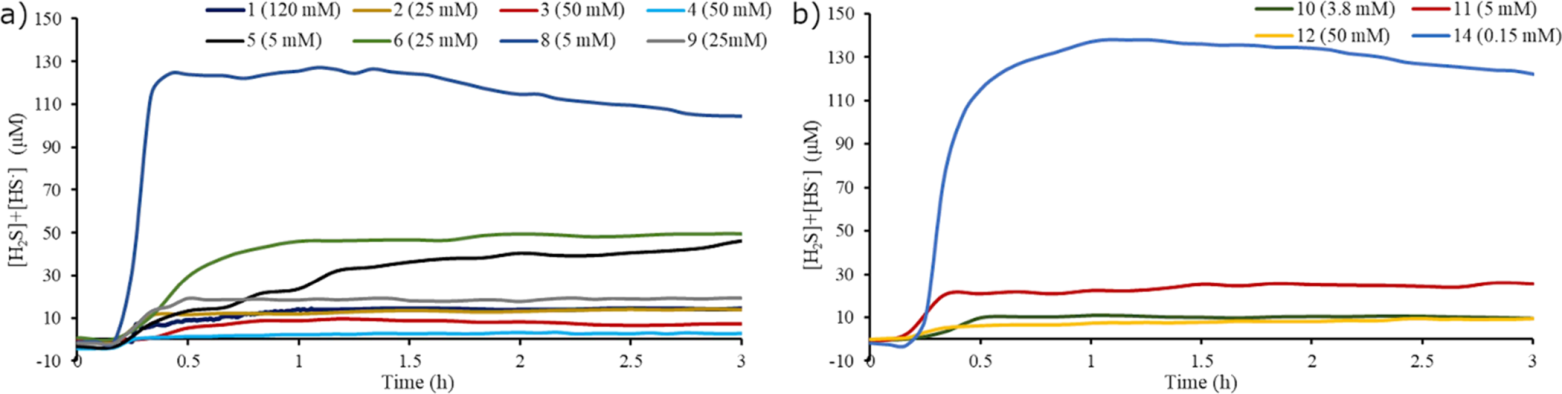
Concentration of sulfide from (a) dialkoxydithiophosphates and (b) disulfidedithiophosphates. The concentration of sulfide was found using H_2_S and pH electrodes.

H_2_S release from the dithiophosphates was measured at room temperature (23–25 °C) using H_2_S and pH electrodes (Figure 7). An advantage of this method over the use of dyes or the methylene blue method is that it can be used to acquire data every few seconds for hours. The electrode measured the concentration of H_2_S, but this can underestimate the release of H_2_S because it has a p*K*_a_ value of 7.0 so a fraction of the H_2_S will be in the form of HS^–^. The fraction of S^2–^ is negligible because of the high p*K*_a_ of HS^–^ which is reported to be in excess of 10. The simultaneous measurement of the concentration of H_2_S and the pH allows the total concentration of sulfide to be calculated. In each of these experiments, the electrodes were immersed in water buffered at a pH value of 6.7 to provide a baseline of no H_2_S release. Next, the dithiophosphates were dissolved in buffered water and added to the buffer with the electrodes. The system was sealed with a rubber stopper while the measurements were taken. H_2_S was detected shortly after the addition of each dithiophosphate and remained fairly constant throughout the measurements.

The data in Figure 7 show that the release of H_2_S from the dithiophosphates mostly followed the trend of the rate constants in Table 2. The highest release of H_2_S was from **8** and **14** that had the fastest rate constants, and the lowest release of H_2_S was from **1** and **4** that had the slowest rate constants. Dithiophosphate **5**, although mostly insoluble at a concentration of 5 mM, released H_2_S at this concentration. The hydrolysis of **5** was not investigated by ^31^P NMR spectroscopy because of its low solubility in water. Dithiophosphates **7** and **13** were also insoluble in water, and when solutions of these chemicals were made at 50 mM they did not show any release of H_2_S (Figures S62 and S63).

### Increased Harvest Yield of Corn Using a Dithiophosphate

Prior work with chemicals that slowly release H_2_S showed that they helped plants survive environmental stressors and increased their harvest yields.^2,11−22,52^ GYY-4137 was shown to increase the harvest yield of lettuce and radishes when grown from seeds to harvest in less than six weeks.^14^ This work was completed in a greenhouse with plants individually planted in growing pots. In an outdoor trial using corn grown individually in potting containers, dibutyldithiophosphate was shown to increase the weight of corn stalks when it was added to the seed at planting and the corn plants were harvested after 4.5 weeks.^11^

**Figure 8:**
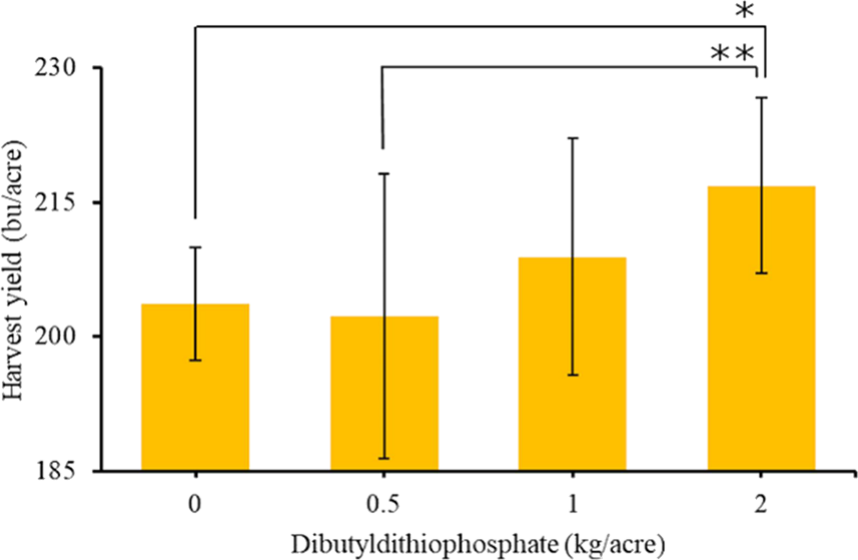
Harvest yields of corn grown with different amounts of dibutyldithiophosphate added per acre are shown. A line connecting two groups indicates statistical significance using the Tukey–Kramer test. *76% confidence interval. **80% confidence interval.

The effect of the slow release of H_2_S on the harvest yield of crops grown outside in fields for months has not been investigated. This represents a large challenge in this area because the amount of rain cannot be controlled, the chemicals that release H_2_S may diffuse away from the seeds, and the chemicals and H_2_S may interact in unknown ways with components of the soil. Despite these challenges, the use of dithiophosphates or other slow-releasing H_2_S chemicals represents a new method to potentially increase the harvest weight of crops. To investigate how dibutyldithiophosphate affects the harvest yield of corn, field trials were completed by an independent, third-party farmer. In these trials, dibutyldithiophosphate was added to a nitrogen-phosphorous-potassium (NPK) starter fertilizer when the seeds were planted. A starter fertilizer of 2-40-28 was applied at a rate of 5 gallons per acre, and dibutyldithiophosphate was added to it to yield an application of 0, 0.5, 1.0, or 2.0 kg per acre of dibutyldithiophosphate. The starter fertilizer with dibutyldithiophosphate was added to the soil in a furrow that connected the seeds that were planted. The planting and harvesting of the corn were performed using state-of-the-art field equipment designed for field trials. Six different plots were fertilized with each loading of dibutyldithiophosphate. Prior to planting the seeds, the soil was fertilized with the NPK fertilizer at loadings for optimal growth of the corn. The corn was harvested, dried, and weighed; the results of the harvest yield are shown in Figure 8.

The results shown in Figure 8 demonstrate that dibutyldithiophosphate can increase the harvest weight of corn even when the dibutyldithiophosphate was only applied once. The biggest effect was observed with a loading of 2 kg/acre of dibutyldithiophosphate and resulted in a 6.4% increase (13.2 bushels per acre) for corn. The harvest yield at 2 kg per acre was higher than the harvest yield grown without dibutyldithiophosphate at a 76% confidence level. At an 80% confidence level, the harvest yield at a 2 kg per acre loading of dibutyldithiophosphate was higher compared to the harvest yield at a loading of 0.5 kg of dibutyldithiophosphate per acre. Approximately 35,000 corn seeds were planted per acre, and a loading of 2.0 kg of dibutyldithiophosphate per acre equates to a dosing of 57 mg of dibutyldithiophosphate per seed. Because the dibutyldithiophosphate was continuously added to the soil in a line connecting the seeds, it is unlikely that each seed adsorbed the full 57 mg of dibutyldithiophosphate. These results demonstrate that the dithiophosphates at very low loadings can have large effects on the harvest yields of corn. Corn seeds from the field trials with 0 and 1 kg/acre of dibutyldithiophosphate added were studied for their nutritional composition (Table S1). These seeds had similar compositions of protein, oil, starch, and projected yield of ethanol.

This article reports the synthesis and characterization of a series of dithiophosphates that showed how their structures affected their rates of hydrolysis, time to complete hydrolysis to yield phosphoric acid, and their rate of release of H_2_S. Several important characteristics of dithiophosphates were discovered including that the fastest hydrolysis belonged to dithiophosphates synthesized using *t*-butanol and 2-mercaptoethanol and that the hydrolysis of dithiophosphates synthesized from thiols was faster than those synthesized from alcohols. These studies were completed in water which is the most relevant solvent to consider for their hydrolysis. The work reported here can be used to design dithiophosphates that release H_2_S at rates and amounts that are desired for different experiments in agriculture. Importantly, all of the dithiophosphates degraded to release phosphoric acid which is common fertilizer and will not result in pollution if used in agriculture.

To investigate the potential for these chemicals to be used in agriculture, dithiophosphate **1** was used to grow corn. Dibutyldithiophosphate was added along a furrow when the seeds were planted using farm equipment that is similar, although smaller, to commercial farm equipment. The corn plants were grown for 146 days after the one-time addition of dibutyldithiophosphate, and an increase in the harvest weight of 6.4% was observed. This experiment and others with chemicals that slowly release H_2_S demonstrate that they can improve the growth and harvest yields of crops and represent a new frontier in this field. Fertilizers are often viewed as a mature field, but the slow release of H_2_S may open up new, unexpected opportunities in this field.

## Supporting Information Available

NMR spectra of the chemicals, kinetic data, and measurement of the slow release of H_2_S using an electrode (PDF)
