# Supplementary material for: Synthesis, Stability, and Kinetics of Hydrogen Sulfide Release of Dithiophosphates
Source: J Agric Food Chem. 2021 Oct 25;69(43):12900–8. doi: 10.1021/acs.jafc.1c04655 (PMC8569798; doi:10.1021/acs.jafc.1c04655)
Supplement: Supplementary file 1 — jf1c04655_si_001.pdf [file jf1c04655_si_001.pdf]

Supporting Information

Synthesis, Stability, and Kinetics of Hydrogen Sulfide Release of Dithiophosphates

Eric M. Brown, Nimesh P. R. Ranasinghe Arachchige, Arjun Paudel, and Ned B. Bowden*
Department of Chemistry
University of Iowa, Iowa City IA, 52242

Ned-bowden@uiowa.edu

Synthesis of dithiophosphate 2. The synthesis of compound 2 was modified from a literature procedure.[1, 2] A mixture of 1,2-ethanediol (0.34 g, 5.36 mmol) and triethylamine (0.58 g, 5.73 mmol) was added slowly over 2 minutes to a mixture of P_2S_5 (0.67 g, 2.98 mmol) and toluene (15 mL). The contents were stirred at 45 °C for 4 h. The pure compound 2 was obtained when the solution dried under reduced pressure and further purified by washing with DCM followed by hexane to give a white solid (77% yield). ^{1}H NMR (300 MHz, D_2O) δ 4.31 (d, 4H), 3.13-3.21 (q, 6H), 1.24 (t, 9H). ^{13}C NMR (300 MHz, $CHCl_3$) δ 68.14, 48.99, 11.36 ^{31}P NMR (300 MHz, D_2O) δ 129.86.

Synthesis of dithiophosphate 3. 1,2-Propanediol (0.45 g, 6 mmol) was added slowly over 2 minutes to a mixture of P_2S_5 (0.65 g, 3 mmol) and toluene (20 mL). The contents were stirred at 90 °C for 12 h. Toluene was removed under reduced pressure, crude compound 3 was cooled in an ice bath, and 8 mL of a 0.75 M potassium hydroxide was added slowly over 2 minutes. The pure compound 3 was obtained when the solution dried under reduced pressure and further purified by flash chromatography using a 20% MeOH in ethyl acetate solvent system to give a white solid (65% yield). ^{1}H NMR (300 MHz, CD_3OD) δ= 4.80-4.85 (m, 1H), 4.54-4.59 (m, 1H), 3.75-3.82 (q, 1H), 1.35-1.37 (d, 3H), ^{31}P NMR (300 MHz, CD_3OD) δ= 129.33. ESI-MS m/z [M+] calculated: 168.9552, found: 168.9541.

Synthesis of dithiophosphate 4. The synthesis of compound 4 was modified from a literature procedure.[2, 3] 1,3-Propanediol (0.45 g, 4.15 mmol) was added slowly over 2 minutes to a mixture of P_2S_5 (0.56g, 2.47 mmol) and toluene (15 mL). The contents were stirred at 90 °C for 16 h. Toluene was removed under reduced pressure, crude compound 4 was cooled in an ice bath, and 8 mL of a 2 M potassium hydroxide was added slowly over 2 minutes. The pure compound 4 was obtained when the solution dried under reduced pressure and further purified by flash chromatography using a 10% MeOH in DCM solvent system to give a white solid (75 % yield). ^{1}H NMR (300 MHz, CD_3OD) δ 4.28-4.37 (dt, 4H), 1.82-1.88 (m, 2H), ^{31}P NMR (300 MHz, CD_3OD) δ 111.60.

Synthesis of dithiophosphate 5. The synthesis of compound 5 was modified from a literature procedure.[4] (+)-Menthol (1.25 g, 8 mmol) was added slowly over 2 minutes to a mixture of P_2S_5 (0.45 g, 2 mmol) and toluene (20 mL). The contents were stirred at 85 °C for 12 h. Toluene was removed under reduced pressure, crude compound 5 was cooled in an ice bath, and 8 mL of a 0.78 M potassium hydroxide was added slowly over 2 minutes. The pure compound 5 was obtained when the solution dried under reduced pressure and further purified by recrystallization from acetone to give a white solid (67 % yield). 1H NMR (300 MHz, CD_3OD,) δ = 4.23-4.46 (m, 2H), 2.54-2.61(m, 2H), 2.31-2.41(m, 2H), 1.62-1.66 (m, 4H), 1.33-1.45 (m, 2), 1.21-1.29 (m, 2H), 0.95-1.12 (m, 4H), 0.83- 0.91 (m, 22H). ^{31}P NMR (300 MHz, CD_3OD) δ = 112.04.

Synthesis of dithiophosphate 6. The synthesis of compound 6 was modified from a literature procedure.[5] 4-Ethylphenol (0.98 g, 8 mmol) was added slowly over 2 minutes to a mixture of P_2S_5 (0.45 g, 2 mmol) and toluene (20 mL). The contents were stirred at 90 °C for 24 h. Toluene was removed under reduced pressure, crude compound 5 was cooled in an ice bath, and 8 mL of a 0.78 M potassium hydroxide was added slowly over 2 minutes. The pure

compound 6 was obtained when the solution dried under reduced pressure and further purified by flash chromatography with ethyl acetate as an eluent to give a white solid (82% yield. ^{1}H NMR (300MHz, DMSO-D$_6$) δ = 7.08–7.12 (m, 4H), 2.46-2.54 (q, 2H), 1.14-1.18 (t, 3H). ^{31}P NMR (DMSO-*d$_6$*) δ= 108.28. **ESI-MS** m/z [M+] calculated: 337.0491, found: 337.0494.

Synthesis of dithiophosphate 7. The synthesis of compound 7 was modified from a literature procedure.[6] (-) Borneol (3.08 g, 20 mmol) was added slowly over 2 minutes to a mixture of P_2S_5 (1.10 g, 4.94 mmol) and toluene (20 mL). The contents were stirred at reflux for 2h. The solution was cooled in an ice bath, and triethylamine (1.01 g, 20 mmol) was added slowly over 2 minutes. The pure compound 7 was obtained when the solution dried under reduced pressure and further purified by recrystallization from acetone to give a white solid (58 % yield). ^{1}H NMR (300 MHz, $CDCl_3$) δ= 4.70-4.71 (t, 2H), 3.26-3.33(q, 6H), 2.20-2.24 (m, 2H), 2.09-2.17(m, 2H), 1.59-1.61 (m, 5H), 1.36-1.40 (t, 11H), 1.27-1.35 (m, 4 H), 0.85-0.96(t, 18H), ^{31}P NMR (300 MHz, $CDCl_3$) δ= 112.31(t).

Synthesis of dithiophosphate 8. The synthesis of compound 8 was modified from a literature procedure.[7] Tert-Butanol (0.71 g, 9.54 mmol) was added slowly over 2 minutes to a mixture of P_2S_5 (0.52 g, 2.34 mmol) and THF (15 mL). The contents were stirred at 45 °C under a nitrogen atmosphere for 5 h. THF was removed under reduced pressure, crude compound 8 was cooled in an ice bath, and 10 mL of a saturated $KHCO_3$ was added slowly over 2 minutes. The pure compound 8 was obtained when the solution dried under reduced pressure to give a white solid (74% yield). ^{1}H NMR (300 MHz, D_2O) δ 1.56 (b). ^{31}P NMR (300 MHz, D_2O) δ 91.36.

Synthesis of dithiophosphate 9. The synthesis of compound 9 was modified from a literature procedure.[8] Iso-Propanol (1.5 mL, 20 mmol) was added slowly over 2 minutes to a mixture of P_2S_5 (0.56 g, 2.5 mmol) and toluene (12 mL). The contents were stirred at 85 °C for 16 h. Toluene was removed under reduced pressure, crude compound 9 was cooled in an ice bath, and 1.2 mL of a 5 M potassium hydroxide was added slowly over 2 minutes. The pure compound 9 was obtained when the solution dried under reduced pressure and further purified by washing with hot toluene to give a white solid (81% yield). ^{1}H NMR (300 MHz, D_2O) δ = 4.69-4.77 (m, 1H), 1.30-1.31 (d, 6H). ^{31}P NMR (300 MHz, D_2O) δ= 107.49.

Synthesis of dithiophosphate 10. 1-Propanethiol (0.59 g. 7.73 mmol) was added slowly over 2 minutes to a mixture of P_2S_5 (0.51 g, 2.24 mmol) and DCM (20 mL). The contents were stirred at reflux for 4 h. DCM was removed under reduced pressure, crude compound 10 was cooled in an ice bath, and triethylamine (0.59 g, 5.73 mmol) was added slowly over 2 minutes The pure compound 10 was obtained when the solution dried under reduced pressure and further purified by flash chromatography using a 10% MeOH in DCM solvent system to give a colorless oily liquid (66 % yield. ^{1}H NMR (300 MHz, CD_3OD) δ 3.18-3.25 (q, 6H), 2.79-2.88 (dt, 4H), 1.65-1.78 (m, 4H), 1.31 (t, 9H), 0.99 (t, 6H). ^{13}C NMR (300 MHz, $CDCl_3$) δ 48.96, 37.11, 29.37, 11.44. ^{31}P NMR (300 MHz, CD_3OD) δ 99.87.

Synthesis of dithiophosphate 11. 1,2-Ethanedithiol (0.45 g, 4.79 mmol) was added slowly over 2 minutes to a mixture of P_2S_5 (0.51 g, 2.25 mmol) and DCM (15 mL). The contents were

stirred at reflux for 3 h. DCM was removed under reduced pressure, crude compound 11 was cooled in an ice bath, and triethylamine (0.59 g, 5.74 mmol) was added slowly over 2 minutes. The pure compound 11 was obtained when the solution dried under reduced pressure and further purified by flash chromatography using a 20% MeOH in DCM solvent system to give a white solid (92 % yield). ^{1}H NMR (300 MHz, DMSO-d_6) δ 3.54 (d, 4H), 3.01-3.13 (q, 6H), 1.17 (t, 9H). ^{13}C NMR (300 MHz, $CHCl_3$) δ 48.94, 31.39, 14.31 ^{31}P NMR (300 MHz, DMSO-d_6) δ 111.73. **ESI-MS** m/z [M+] calculated: 186.8938, found: 186.8936.

Synthesis of dithiophosphate 12. The synthesis of compound 12 was modified from a literature procedure.[2] 1,3-Propanedithiol (0.43 g, 4.52 mmol) was added slowly over 2 minutes to a mixture of P_2S_5 (0.51 g, 2.25 mmol) and DCM (15 mL). The contents were stirred at reflux for 3 h. DCM was removed under reduced pressure, crude compound 12 was cooled in an ice bath, and triethylamine (0.59 g, 5.74 mmol) was added slowly over 2 minutes. The pure compound 12 was obtained when the solution dried under reduced pressure and further purified by flash chromatography using a 30% MeOH in DCM solvent system to give a white solid (93 % yield). ^{1}H NMR (400 MHz, DMSO-d_6) δ 3.07-3.15 (m, 10H), 1.83-1.91 (p, 2H), 1.17 (t, 9H). ^{13}C NMR (300 MHz, $CHCl_3$) δ 48.96, 37.06, 29.37, 11.44 ^{31}P NMR (300 MHz, DMSO-d_6) δ 78.62. **ESI-MS** m/z [M+] calculated: 200.9095, found: 200.9090.

Synthesis of dithiophosphate 13. The synthesis of compound 13 was modified from a literature procedure.[9] Thiophenol (1.08 g, 9.98 mmol) was added slowly over 2 minutes to a mixture of P_2S_5 (0.51 g, 2.26 mmol) and DCM (10 mL). The contents were stirred at reflux for 5 h. DCM was removed under reduced pressure, crude compound 13 was cooled in an ice bath, and triethylamine (0.58 g, 5.73 mmol) was added slowly over 2 minutes. The pure compound 13 was obtained when the solution dried under reduced pressure and further purified by flash chromatography using a 20% MeOH in DCM solvent system to give a white solid (82 % yield). ^{1}H NMR (300 MHz, CD_3OD) δ 7.63-7.67 (m, 4H), 7.29-7.34 (m, 6H), 3.16-3.23 (q, 6H), 1.29 (t, 9H), ^{13}C NMR (300 MHz, CD_3OD) δ 131.81, 129.28, 124.14, 47.60, 9.41. ^{31}P NMR (300 MHz, CD_3OD) δ 100.70. **ESI-MS** m/z [M+] calculated: 312.9408, found: 312.9412.

Synthesis of dithiophosphate 14. Synthesis of chemical 14 was modified from a literature procedure.[2] 2-Mercaptoethanol (0.45 g, 5.83 mmol) was added slowly over 2 minutes to a mixture of P_2S_5 (0.54 g, 2.47 mmol) and DCM (15 mL). The contents were stirred at reflux for 3 h. DCM was removed under reduced pressure, crude compound 14 was cooled in an ice bath, and triethylamine (0.28 g, 2.86 mmol) was added slowly over 2 minutes. The pure compound 14 was obtained when the solution dried under reduced pressure to give a colorless oily liquid (86% yield). ^{1}H NMR (300 MHz, CD_3OD) δ 4.01-4.09 (dt, 2H), 3.21-3.28 (q, 6H), 2.74 (t, 2H), 1.33 (t, 9H). ^{13}C NMR (300 MHz, $CHCl_3$) δ 70.18, 48.88, 27.70, 11.46 ^{31}P NMR (300 MHz, CD_3OD) δ 112.39.

The hydrolysis of 2 at room temperature in 90% H_2O/D_2O measured by ^{31}P NMR spectroscopy. Dithiophosphate **2** (81.2 mg, 0.32 mmol) was dissolved in 1 mL of 90 % H_2O/D_2O, yielding a 0.21 M solution. ^{31}P NMR spectra (300 MHz, 90 % H_2O/D_2O) were collected at day 0 and day 30.

The hydrolysis of 3 at room temperature in 90% H_2O/D_2O measured by ^{31}P NMR spectroscopy. Dithiophosphate **3** (10.4 mg, 0.05 mmol) was dissolved in 1 mL of 90 % H_2O/D_2O, yielding a 50 mM solution. ^{31}P NMR spectra (300 MHz, 90 % H_2O/D_2O) were collected at day 0 and day 30.

The hydrolysis of chemical 4 at room temperature in 90% H_2O/D_2O measured by ^{31}P NMR spectroscopy. Dithiophosphate **4** (80.0 mg, 0.387 mmol) was dissolved in 1.5 mL of 90 % H_2O/D_2O, yielding a 0.25 M solution. ^{31}P NMR spectra (300 MHz, 90 % H_2O/D_2O) were collected at day 0 and day 30.

The hydrolysis of chemical 6 at room temperature in 90% H_2O/D_2O measured by ^{31}P NMR spectroscopy. Dithiophosphate **6** (18.8 mg, 0.05 mmol) was dissolved in 1 mL of 90 % H_2O/D_2O, yielding a 50 mM solution. ^{31}P NMR spectra (300 MHz, 90 % H_2O/D_2O) were collected at day 0 and day 30.

The hydrolysis of chemical 8 at room temperature in 90% H_2O/D_2O measured by ^{31}P NMR spectroscopy. Dithiophosphate **8** (130 mg, 0.50 mmol) was dissolved in 1.5 mL of 90 % H_2O/D_2O, yielding a 0.34 M solution. ^{31}P NMR spectra (300 MHz, 90 % H_2O/D_2O) were collected at day 0 and day 30.

The hydrolysis of chemical 9 at room temperature in 90% H_2O/D_2O measured by ^{31}P NMR spectroscopy. Dithiophosphate **9** (74.0 mg, 0.29 mmol) was dissolved in 1 mL of 90 % H_2O/D_2O, yielding a 0.29 M solution. ^{31}P NMR spectra (300 MHz, 90 % H_2O/D_2O) were collected at day 0 and day 30.

The hydrolysis of chemical 10 at room temperature in 90% H_2O/D_2O measured by ^{31}P NMR spectroscopy. Dithiophosphate **10** (1.95 mg, 5.6 µmol) was dissolved in 1.5 mL of 90 % H_2O/D_2O, yielding a 3.75 mM solution. ^{31}P NMR spectra (300 MHz, 90 % H_2O/D_2O) were collected at day 0 and day 30.

The hydrolysis of chemical 11 at room temperature in 90% H_2O/D_2O measured by ^{31}P NMR spectroscopy. Dithiophosphate **11** (105 mg, 0.36 mmol) was dissolved in 1.5 mL of 90 % H_2O/D_2O, yielding a 0.25 M solution. ^{31}P NMR spectra (300 MHz, 90 % H_2O/D_2O) were collected at day 0 and day 30.

The hydrolysis of chemical 12 at room temperature in 90% H_2O/D_2O measured by ^{31}P NMR spectroscopy. Dithiophosphate **12** (102 mg, 0.33 mmol) was dissolved in 1.5 mL of 90 % H_2O/D_2O, yielding a 0.22 M solution. ^{31}P NMR spectra (300 MHz, 90 % H_2O/D_2O) were collected at day 0 and day 30.

The hydrolysis of chemical 13 at room temperature in 3:1 DMSO-*d_6*:H_2O measured by ^{31}P NMR spectroscopy. Dithiophosphate **13** (103 mg, 0.25 mmol) was dissolved in 1 mL of 3:1 DMSO-d6:H_2O, yielding a 0.25 M solution. ^{31}P NMR spectra (300 MHz, 90 % H_2O/D_2O) were collected at day 0 and day 30.

The hydrolysis of chemical 14 at room temperature in 90% H_2O/D_2O measured by ^{31}P NMR spectroscopy. Dithiophosphate **14** (100 mg, 0.37 mmol) was dissolved in 1.5 mL of 90%

H_2O/D_2O, yielding a 0.25 M solution. ^{31}P NMR spectra (300 MHz, 90 % H_2O/D_2O) were collected at day 0 and day 30.

Investigation of rates of hydrolysis of dithiophosphate salts at 85 °C by ^{31}P NMR spectroscopy.

Dithiophosphate **2** (98.9 mg, 0.38 mmol) was dissolved in 1.5 mL of 90% H_2O/D_2O, yielding a 0.26 M solution. The solution was added to an NMR tube and placed in an 85 °C oil bath. ^{31}P NMR spectra (300 MHz) were taken periodically to track degradation.

Dithiophosphate **3** (10.4 mg, 0.05 mmol) was dissolved in 1 mL of 90% H_2O/D_2O, yielding a 50 mM solution. The solution was added to an NMR tube and placed in an 85 °C oil bath. ^{31}P NMR spectra (300 MHz) were taken periodically to track degradation.

Dithiophosphate **4** (80.1 mg, 0.38 mmol) was dissolved in 1.5 mL of 90% H_2O/D_2O, yielding a 0.26 M solution. The solution was added to an NMR tube and placed in an 85 °C oil bath. ^{31}P NMR spectra (300 MHz) were taken periodically to track degradation.

Dithiophosphate **6** (18.8 mg, 0.05 mmol) was dissolved in 1 mL 90 % H_2O/D_2O, yielding a 50 mM solution. The solution was added to an NMR tube and placed in an 85 °C oil bath. ^{31}P NMR spectra (300 MHz) were taken periodically to track degradation.

Dithiophosphate **8** (132 mg, 0.51 mmol) was dissolved in 1.5 mL of 90% H_2O/D_2O, yielding a 0.34 M solution. The solution was added to an NMR tube and placed in an 85 °C oil bath. ^{31}P NMR spectra (300 MHz) were taken periodically to track degradation.

Dithiophosphate **9** (0.074 mg, 0.29 mmol) was dissolved in 1 mL of 90% H_2O/D_2O, yielding a 0.29 M solution. The solution was added to an NMR tube and placed in an 85 °C oil bath. ^{31}P NMR spectra (300 MHz) were taken periodically to track degradation.

Dithiophosphate **10** (1.95 mg, 5.61 µmol) was dissolved in 1.5 mL of 90% H_2O/D_2O, yielding 3.75 mM solution. The solution was added to an NMR tube and placed in an 85 °C oil bath. ^{31}P NMR spectra (300 MHz) were taken periodically to track degradation.

Dithiophosphate **11** (102 mg, 0.35 mmol) was dissolved in 1.5 mL of 90% H_2O/D_2O, yielding a 0.24 M solution. The solution was added to an NMR tube and placed in an 85 °C oil bath. ^{31}P NMR spectra (300 MHz) were taken periodically to track degradation.

Dithiophosphate **12** (100 mg, 0.33 mmol) was dissolved in 1.4 mL of 90% H_2O/D_2O, yielding a 0.24 M solution. The solution was added to an NMR tube and placed in an 85 °C oil bath. ^{31}P NMR spectra (300 MHz) were taken periodically to track degradation.

Dithiophosphate **13** (103 mg, 0.25 mmol) was dissolved in 1 mL of 90% H_2O/D_2O, yielding a 0.25 M solution. The solution was added to an NMR tube and placed in an 85 °C oil bath. ^{31}P NMR spectra (300 MHz) were taken periodically to track degradation.

Dithiophosphate **14** (101 mg, 0.36 mmol) was dissolved in 1.5 mL of 90% H_2O/D_2O, yielding a 0.25 M solution. The solution was added to an NMR tube and placed in an 85 °C oil bath. ^{31}P NMR spectra (300 MHz) were taken periodically to track degradation.

Investigation of rates of hydrolysis of 8 and 14 at room temperature by ^{31}P NMR spectroscopy.

Dithiophosphate **8** (132 mg, 0.51 mmol) was dissolved in 1.5 mL of 90% H_2O/D_2O, yielding a 0.34 M solution. The solution was added to an NMR tube. ^{31}P NMR spectra (300 MHz) were taken periodically to track degradation.

Dithiophosphate **14** (91.8 mg, 0.33 mmol) was dissolved in 1.5 mL of 90% H_2O/D_2O, yielding a 0.22 M solution. The solution was added to an NMR tube. ^{31}P NMR spectra (300 MHz) were taken periodically to track degradation.

Investigation of hydrolysis of 8, 10, 11, and 14 at multiple temperatures by ^{31}P NMR spectroscopy to obtain $\Delta H^{\ddagger}$ and $\Delta S^{\ddagger}$.

Dithiophosphate **8** (132 mg, 0.51 mmol) was dissolved in 1.5 mL of 90% H_2O/D_2O, yielding a 0.34 M solution. The solution was added to an NMR tube and placed in a 45 °C oil bath. ^{31}P NMR spectra (300 MHz) were taken periodically to track degradation.

Dithiophosphate **8** (132 mg, 0.51 mmol) was dissolved in 1.5 mL of 90% H_2O/D_2O, yielding a 0.34 M solution. The solution was added to an NMR tube and placed in a 60 °C oil bath. ^{31}P NMR spectra (300 MHz) were taken periodically to track degradation.

Dithiophosphate **10** (1.95 mg, 5.61 μmol) was dissolved in 1.5 mL of 90% H_2O/D_2O, yielding 3.75 mM solution. The solution was added to an NMR tube and placed in a 60 °C oil bath. ^{31}P NMR spectra (300 MHz) were taken periodically to track degradation.

Dithiophosphate **10** (1.95 mg, 5.61 μmol) was dissolved in 1.5 mL of 90% H_2O/D_2O, yielding 3.75 mM solution. The solution was added to an NMR tube and placed in a 70 °C oil bath. ^{31}P NMR spectra (300 MHz) were taken periodically to track degradation.

Dithiophosphate **11** (102 mg, 0.35 mmol) was dissolved in 1.5 mL of 90% H_2O/D_2O, yielding a 0.24 M solution. The solution was added to an NMR tube and placed in a 60 °C oil bath. ^{31}P NMR spectra (300 MHz) were taken periodically to track degradation.

Dithiophosphate **11** (102 mg, 0.35 mmol) was dissolved in 1.5 mL of 90% H_2O/D_2O, yielding a 0.24 M solution. The solution was added to an NMR tube and placed in a 70 °C oil bath. ^{31}P NMR spectra (300 MHz) were taken periodically to track degradation.

Dithiophosphate **14** (101 mg, 0.36 mmol) was dissolved in 1.5 mL of 90% H_2O/D_2O, yielding a 0.25 M solution. The solution was added to an NMR tube and placed in a 45 °C oil bath. ^{31}P NMR spectra (300 MHz) were taken periodically to track degradation.

Dithiophosphate **14** (101 mg, 0.36 mmol) was dissolved in 1.5 mL of 90% H_2O/D_2O, yielding a 0.25 M solution. The solution was added to an NMR tube and placed in a 60 °C oil bath. ^{31}P NMR spectra (300 MHz) were taken periodically to track degradation.

Nutritional test for corn

Table S1. The composition of harvested corn seeds when 0 and 1 kg/acre of dibutyldithiophosphate were applied.

Amount of dithiophosphate applied (kg/acre)	Moisture (%)	Protein (%)	Oil (%)	Starch (%)	Density (g/cc)	Projected yield of ethanol (gallon/bushel)
0	11.8	7.0	3.2	61.6	1.260	2.8
1	12.2	6.8	3.3	61.8	1.270	2.8

NMR spectra of the dithiophosphates are shown below.

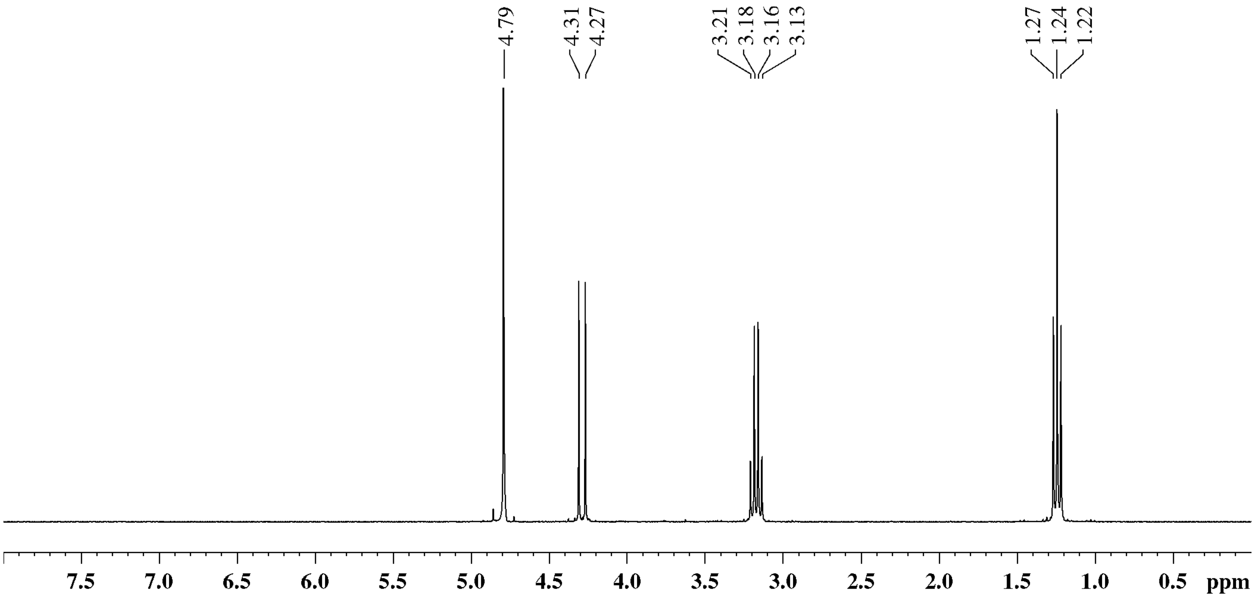

Figure S1. ^{1}H NMR spectrum of **2** in D_2O.

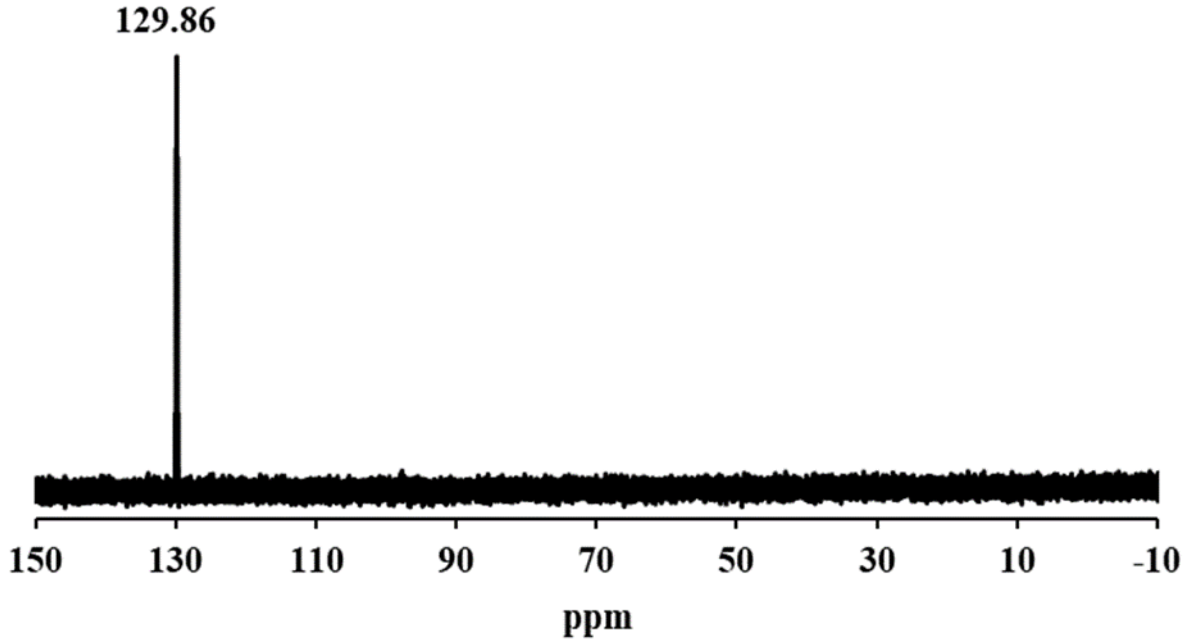

Figure S2. ^{31}P NMR spectrum of **2** in D_2O.

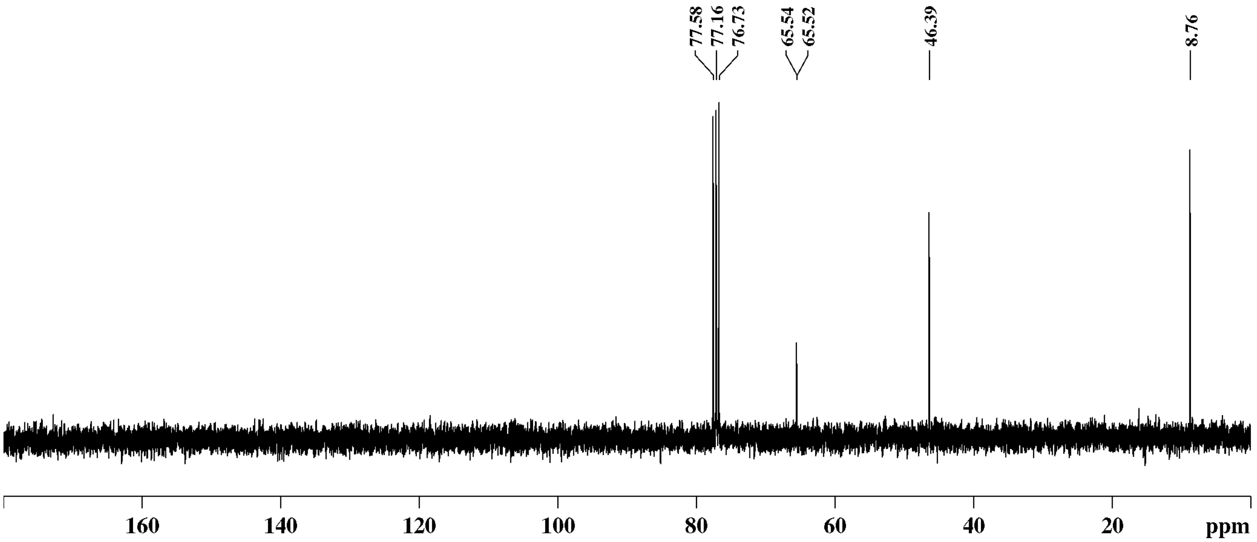

Figure S3. ^{13}C NMR spectrum of **2** in $CDCl_3$.

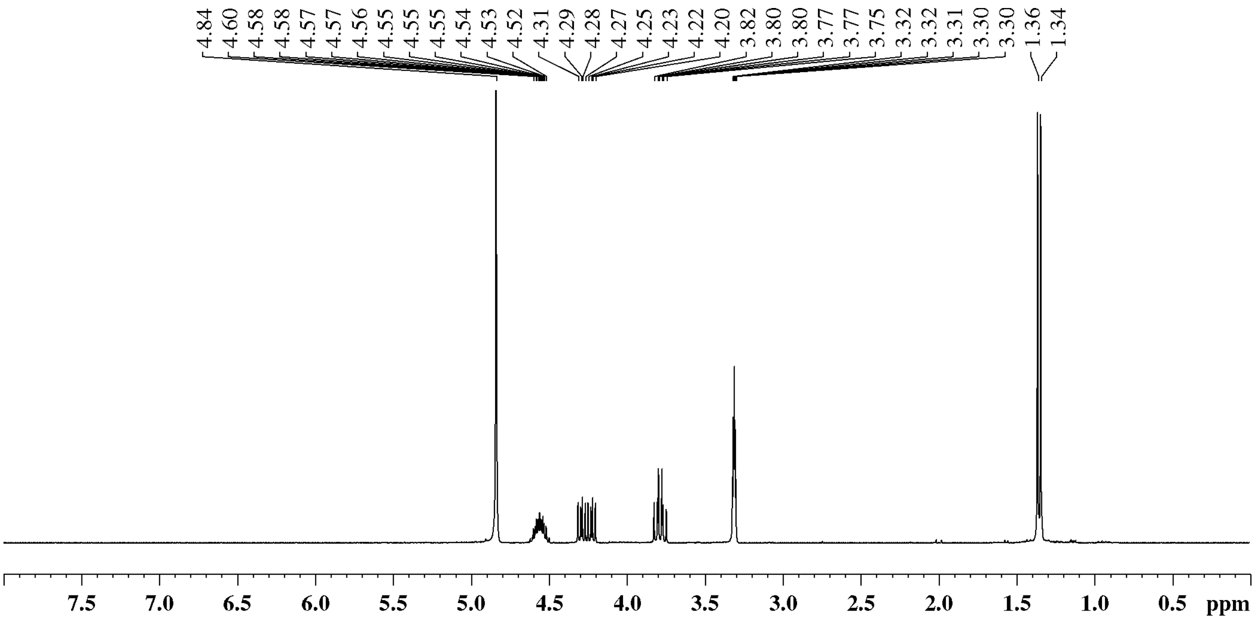

Figure S4. ^{1}H NMR spectrum of **3** in CD_3OD.

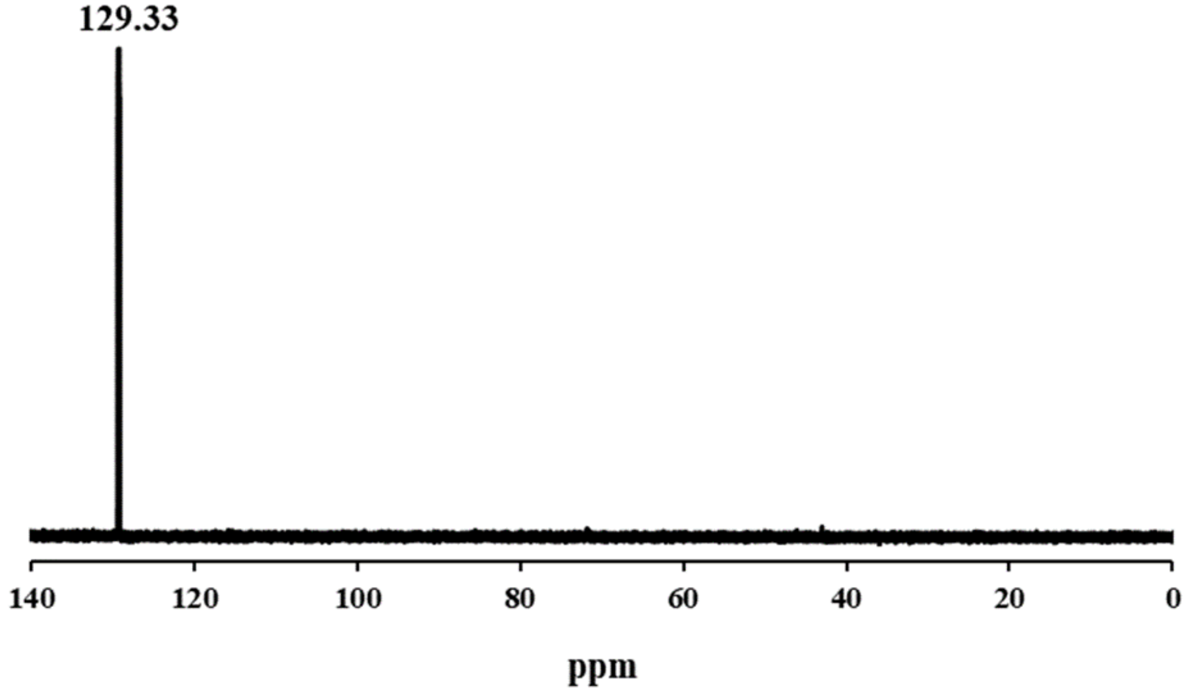

Figure S5. ^{31}P NMR spectrum of **3** in CD_3OD.

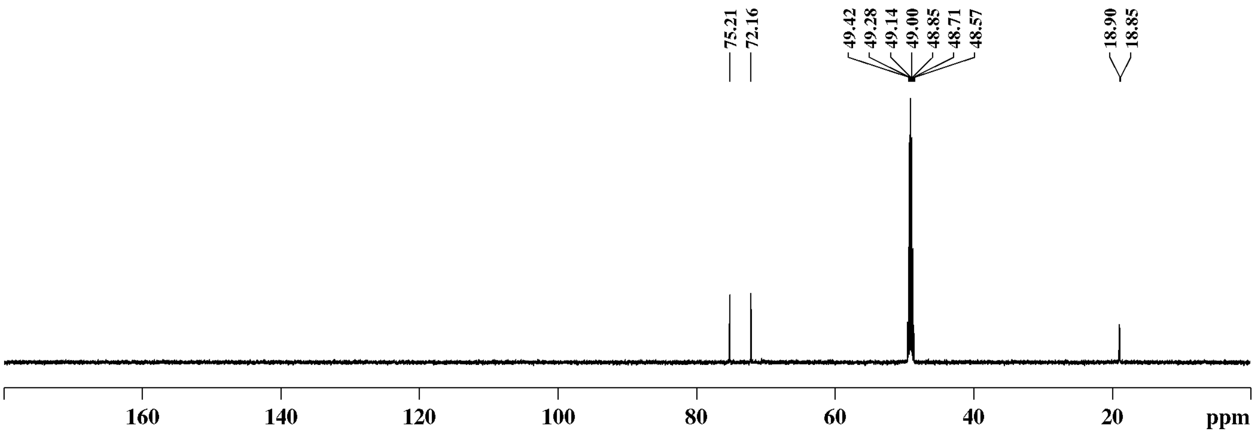

Figure S6. ^{13}C NMR spectrum of **3** in CD_3OD.

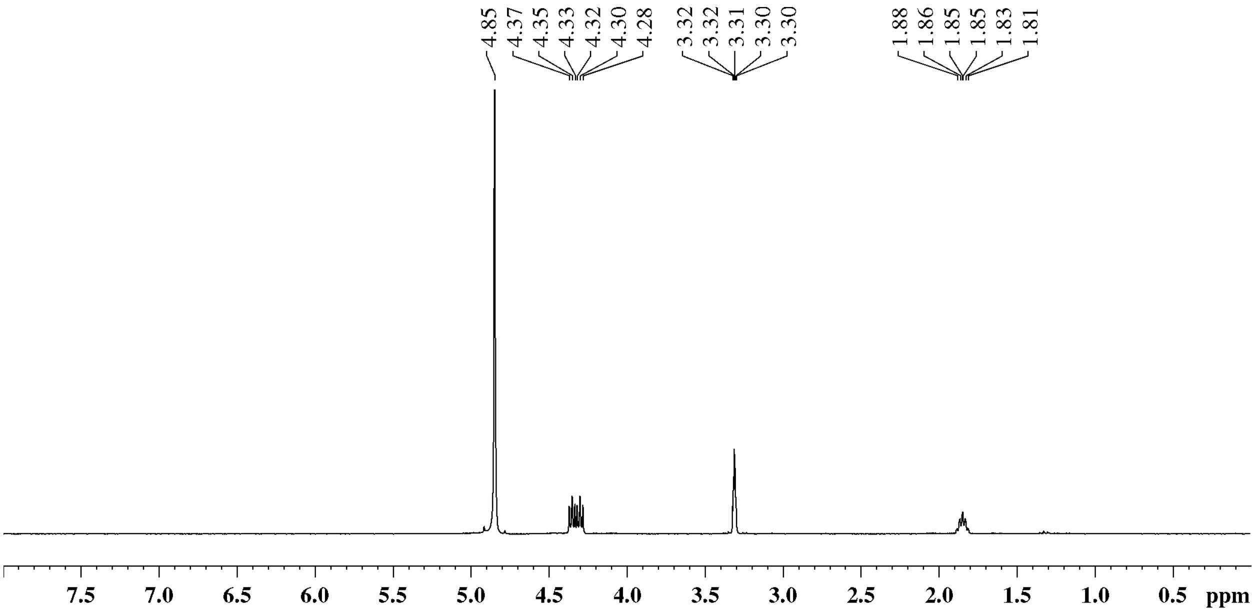

Figure S7. ^{1}H NMR spectrum of **4** in CD_3OD.

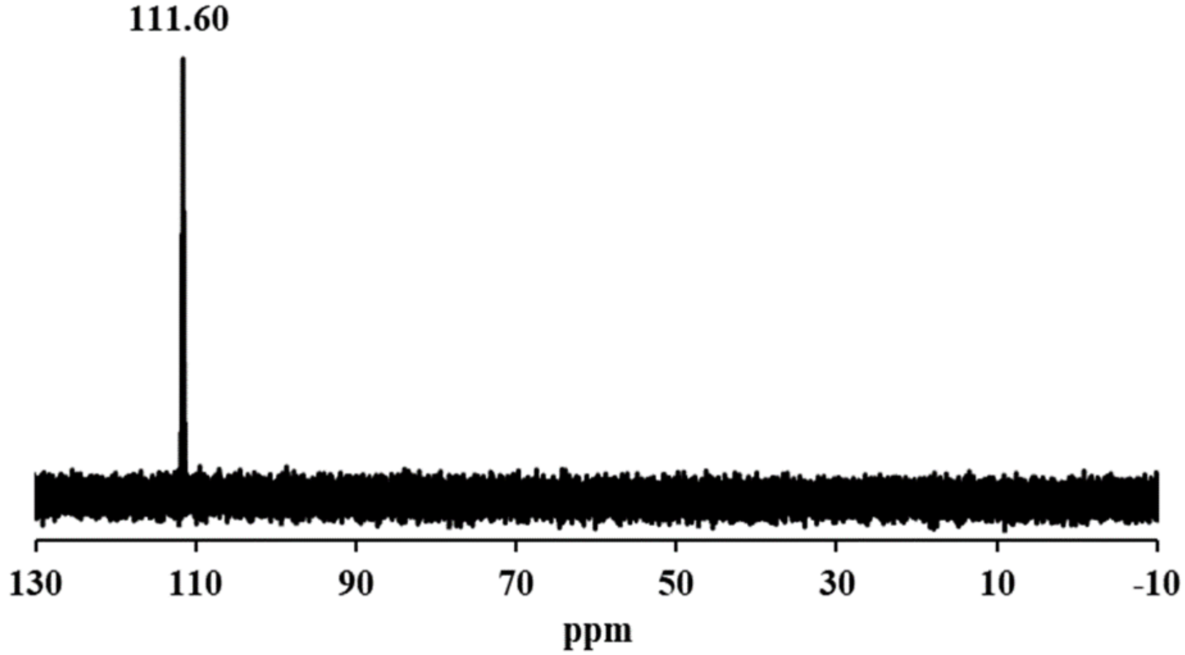

Figure S8. ^{31}P NMR spectrum of **4** in CD_3OD.

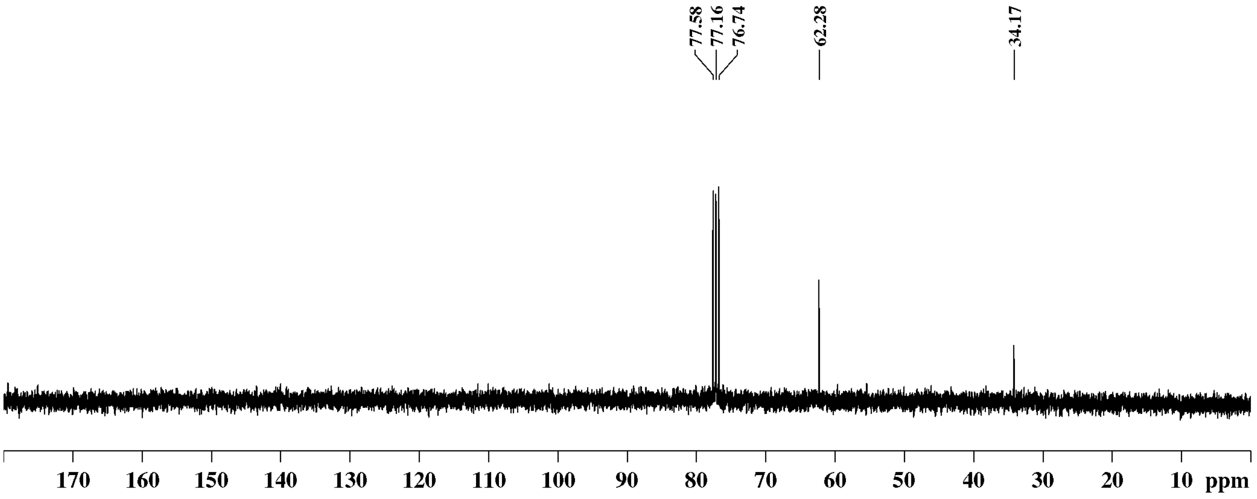

Figure S9. ^{13}C NMR spectrum of **4** in $CDCl_3$.

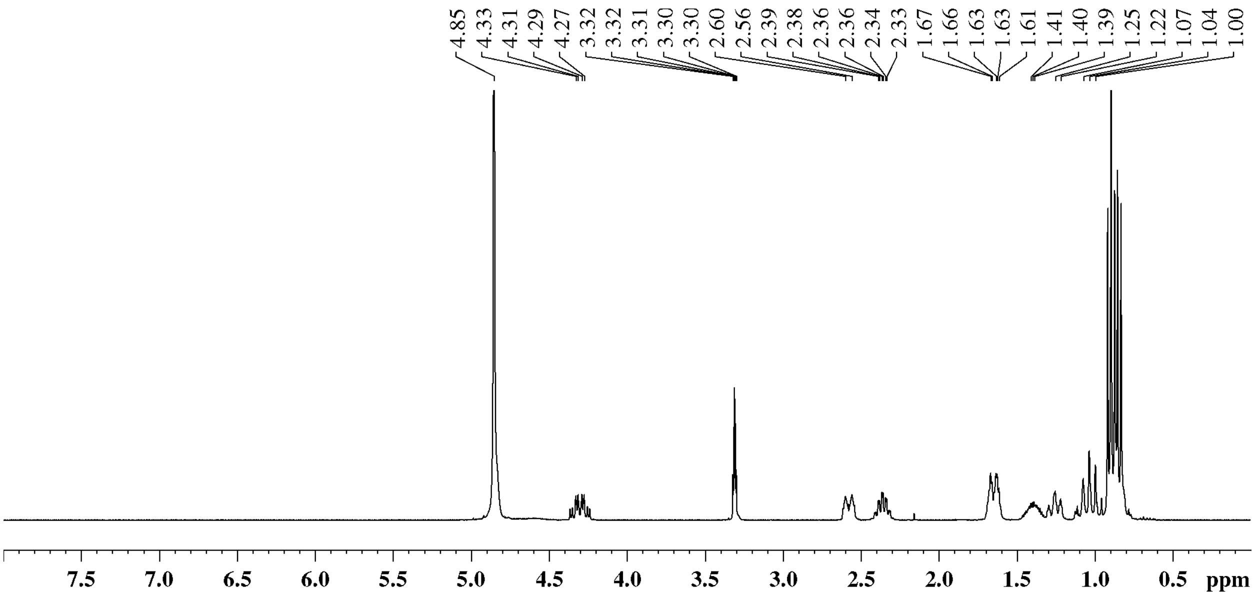

Figure S10. ^{1}H NMR spectrum of **5** in CD_3OD.

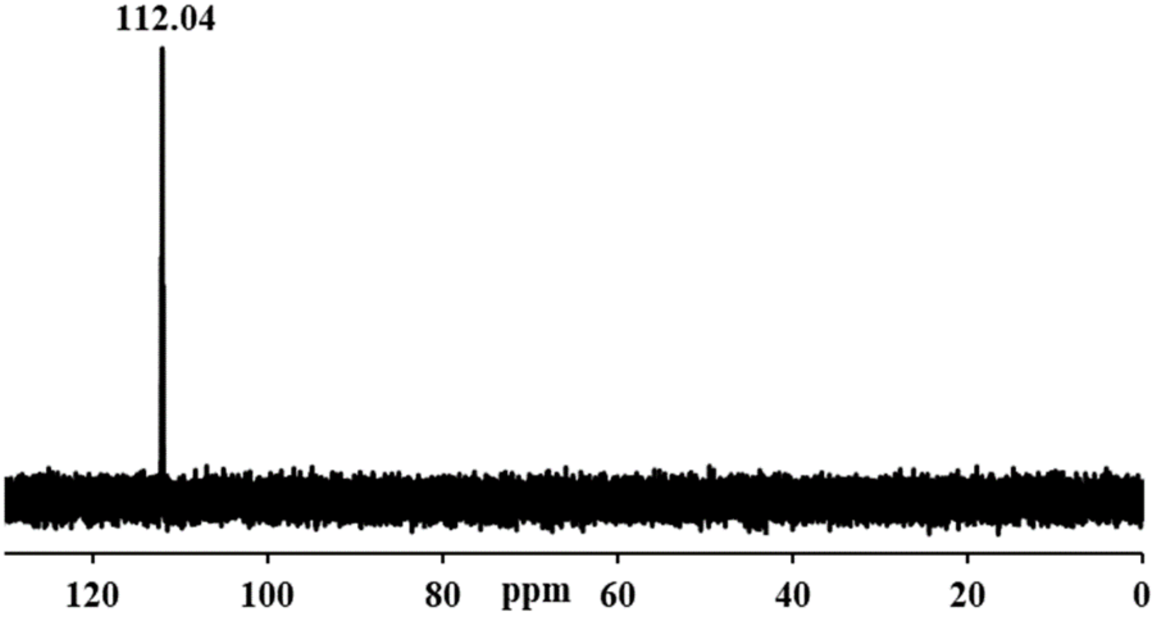

Figure S11. ^{31}P NMR spectrum of **5** in CD_3OD.

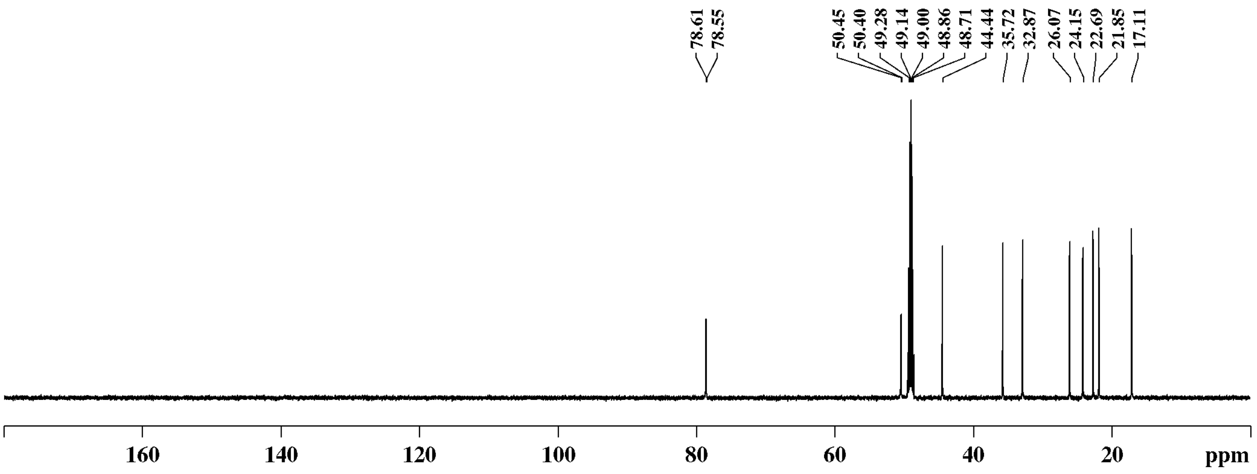

Figure S12. ^{13}C NMR spectrum of **5** in CD_3OD.

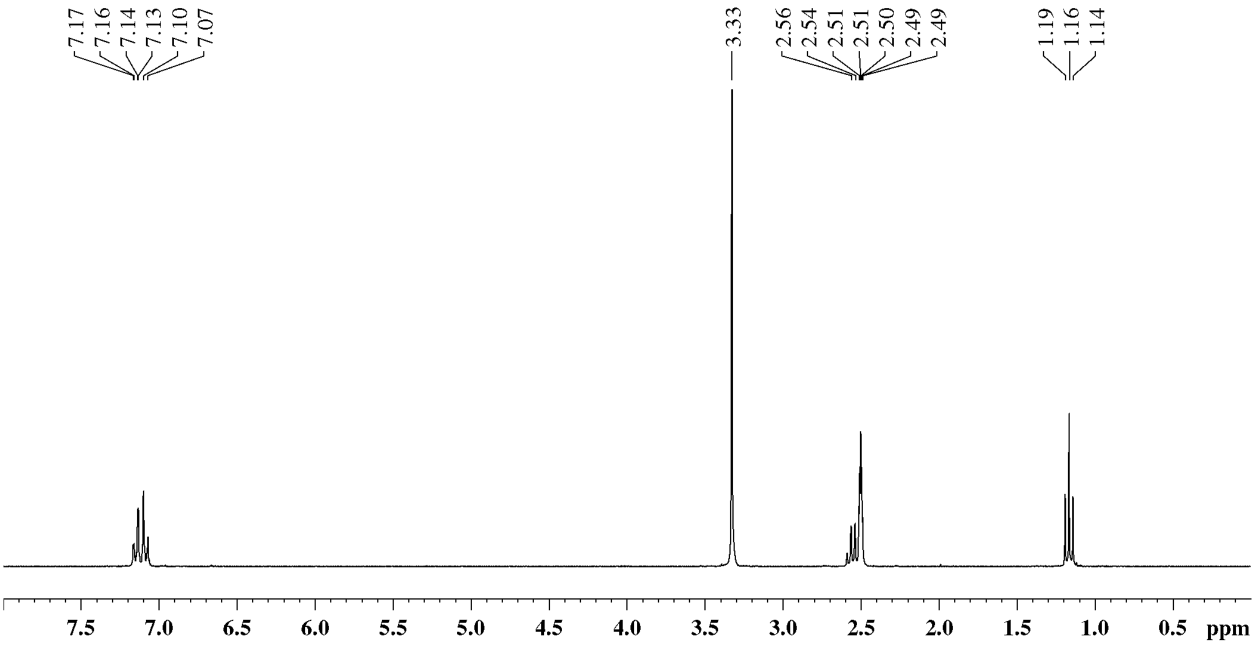

Figure S13. ^{1}H NMR spectrum of **6** in DMSO-d_6.

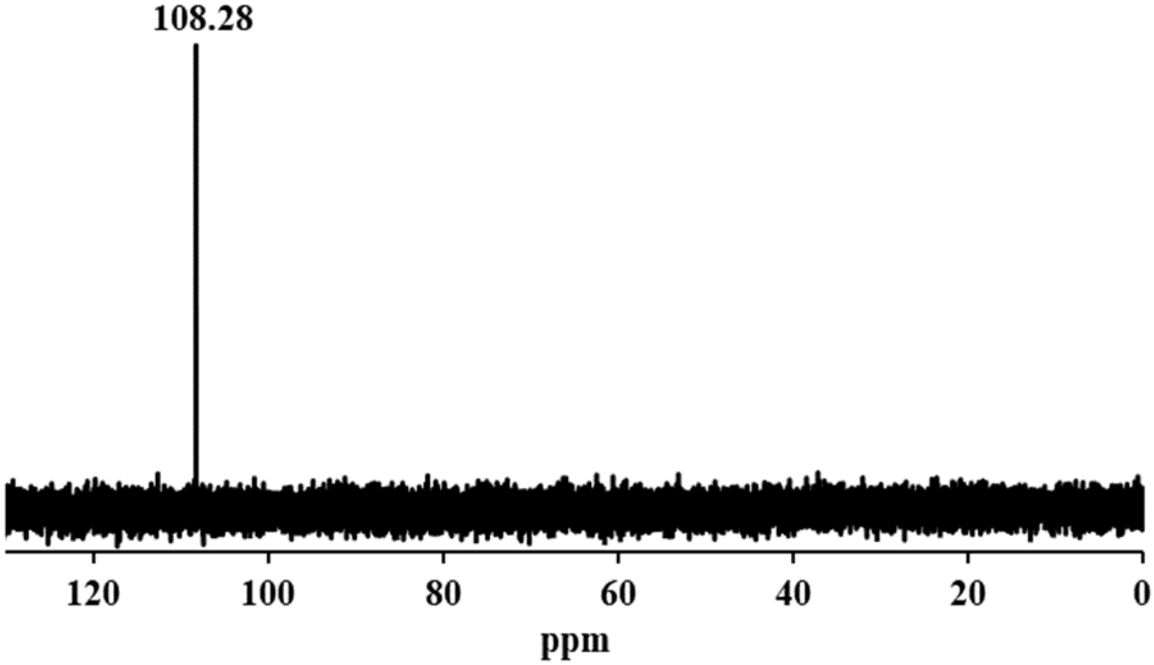

Figure S14. ^{31}P NMR spectrum of **6** in DMSO-d_6.

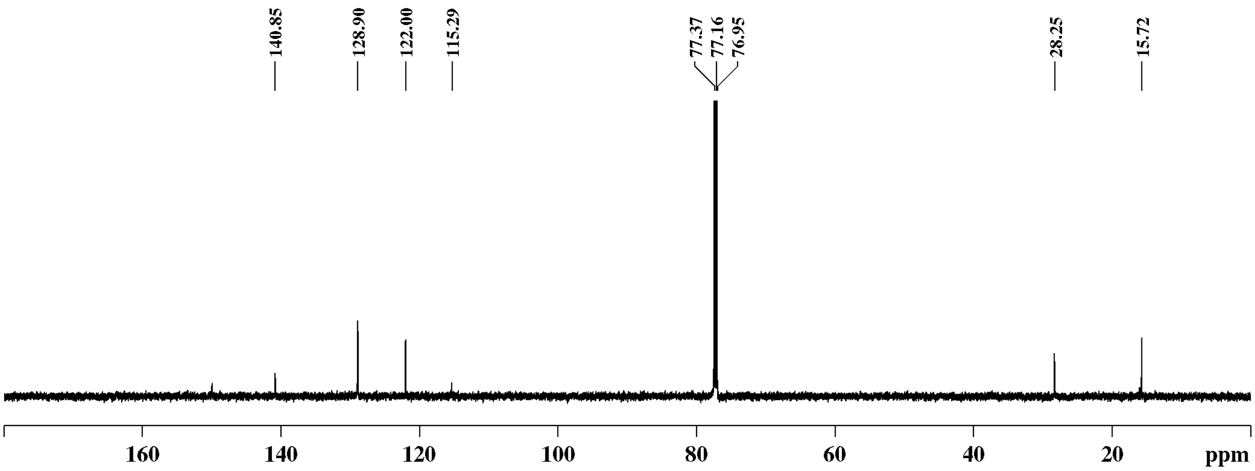

Figure S15. ^{13}C NMR spectrum of **6** in $CDCl_3$.

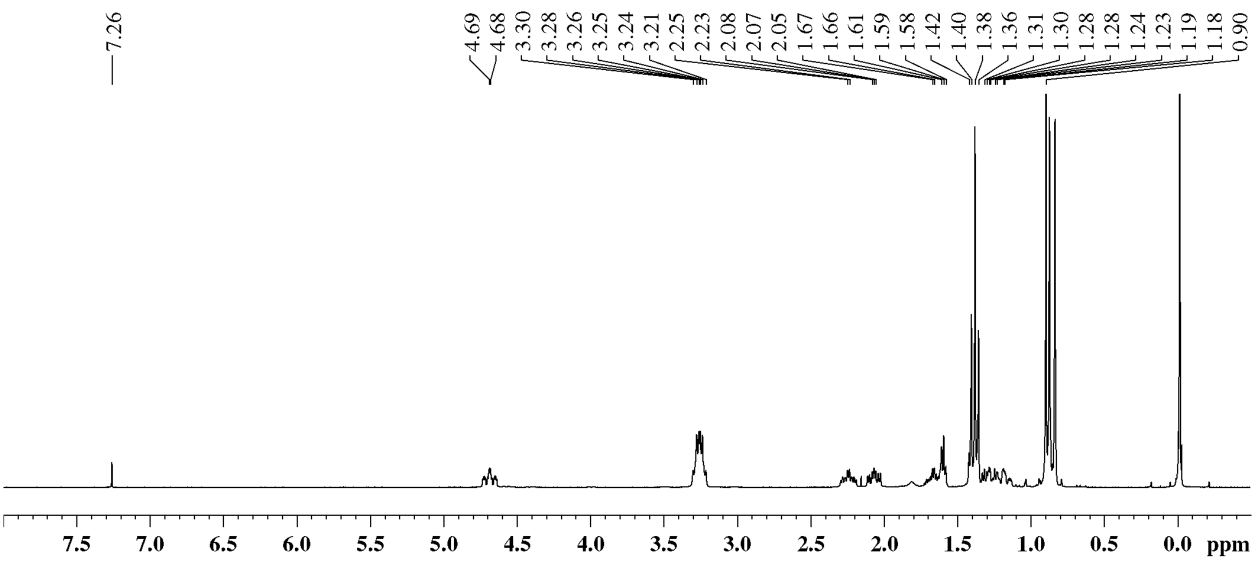

Figure S16. ^{1}H NMR spectrum of **7** in $CDCl_3$.

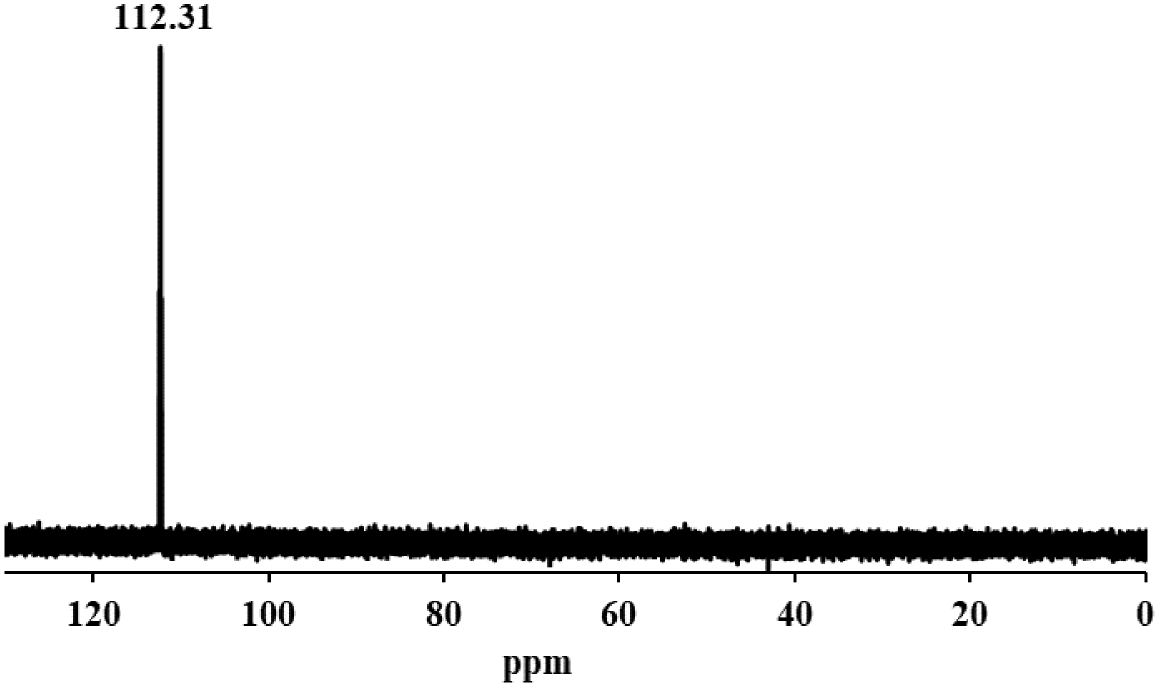

Figure S17. ^{31}P NMR spectrum of **7** in $CDCl_3$.

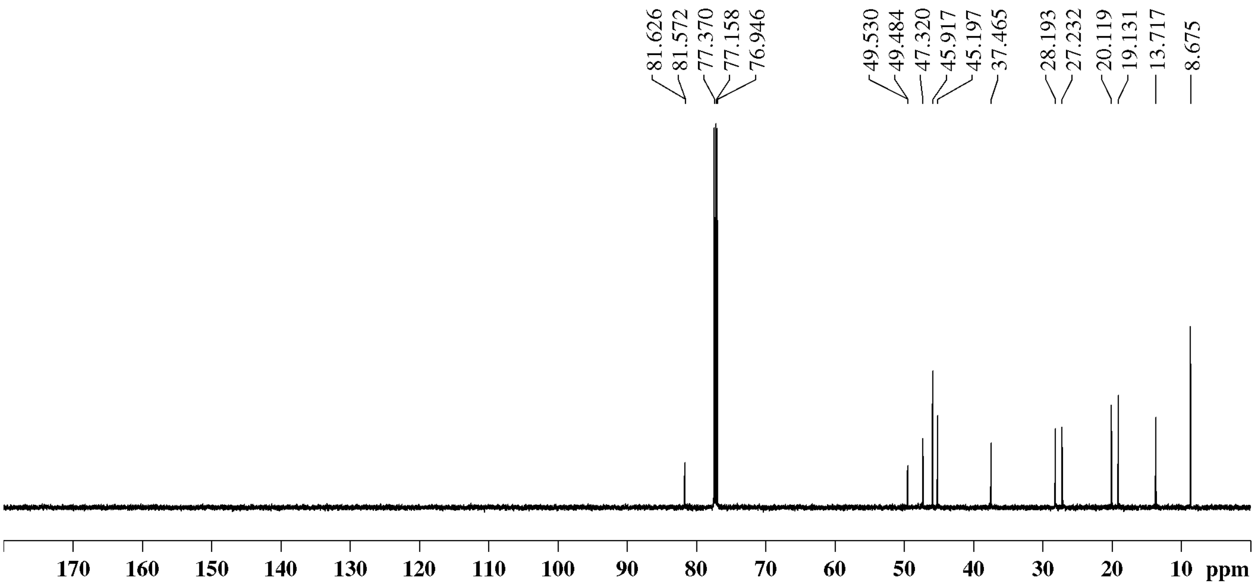

Figure S18. ^{13}C NMR spectrum of **7** in $CDCl_3$.

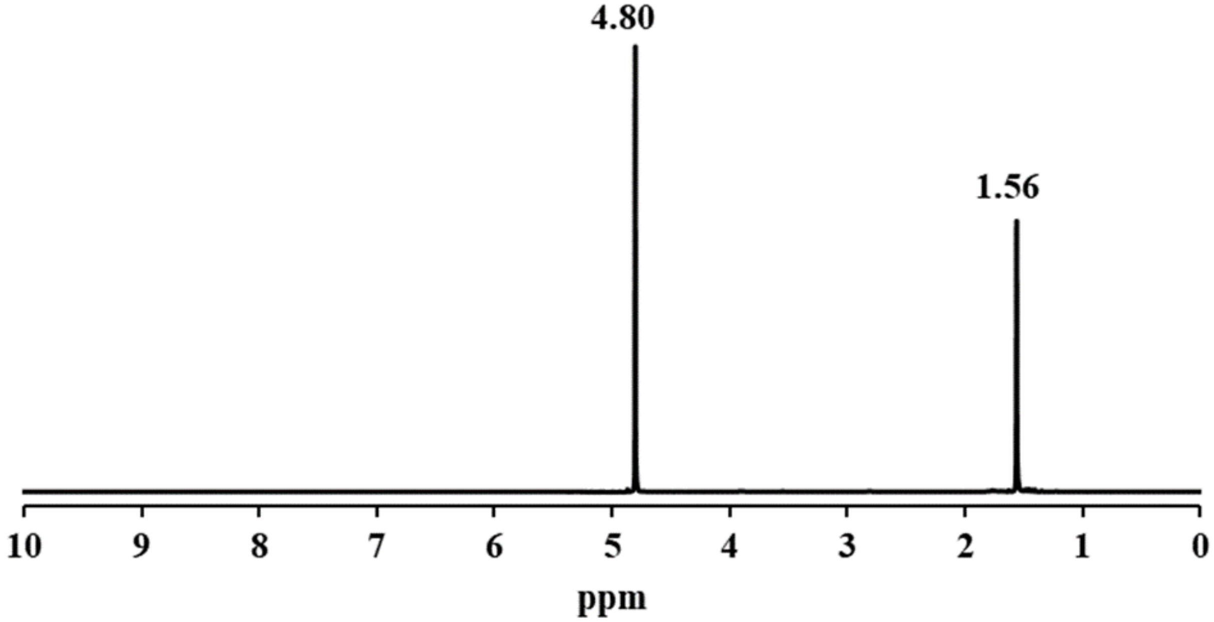

Figure S19. ^{1}H NMR spectrum of **8** in D_2O.

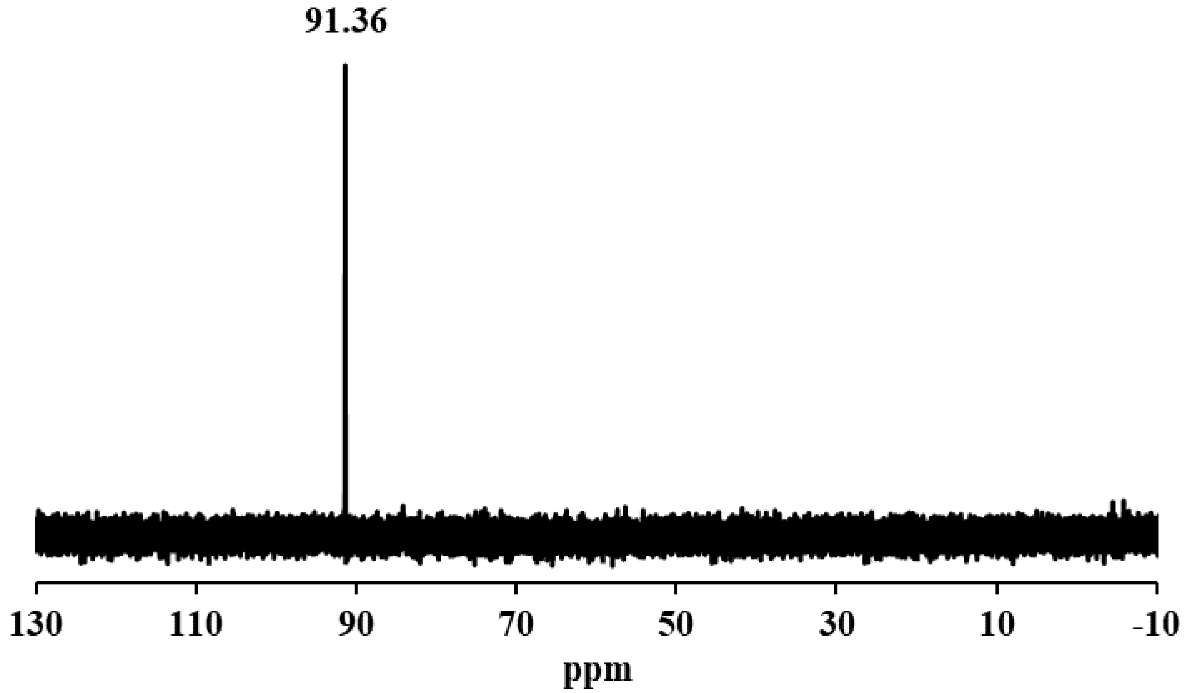

Figure S20. ^{31}P NMR spectrum of **8** in D_2O.

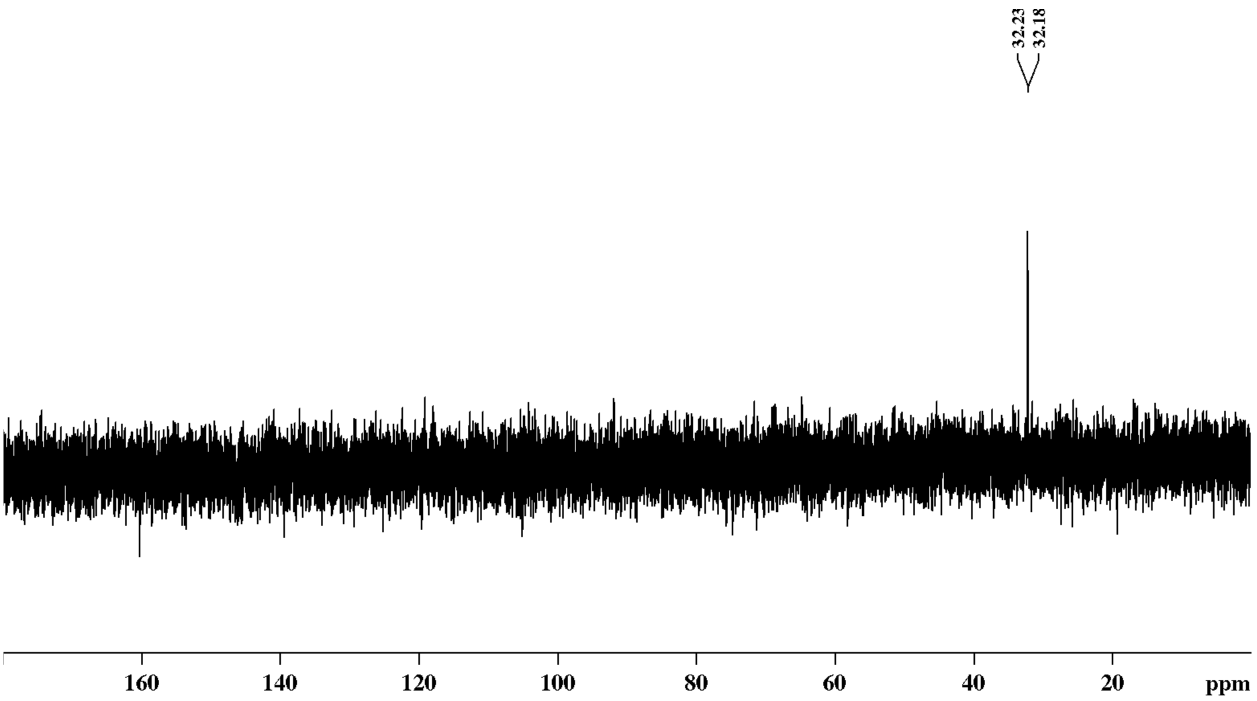

Figure S21. ^{13}C NMR spectrum of **8** in D_2O.

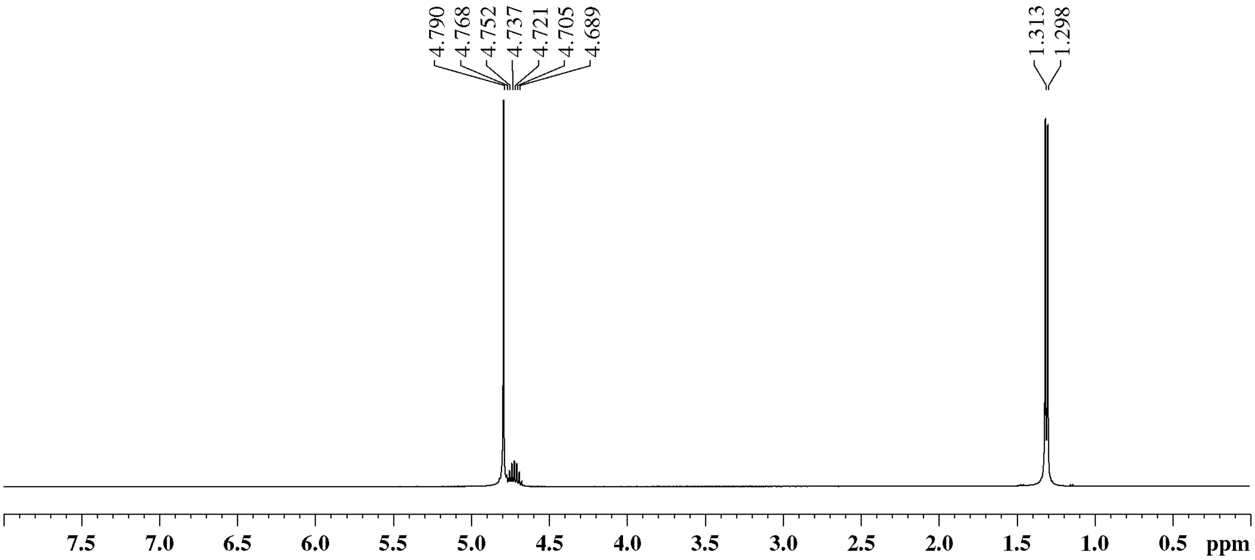

Figure S22. ^{1}H NMR spectrum of **9** in D_2O.

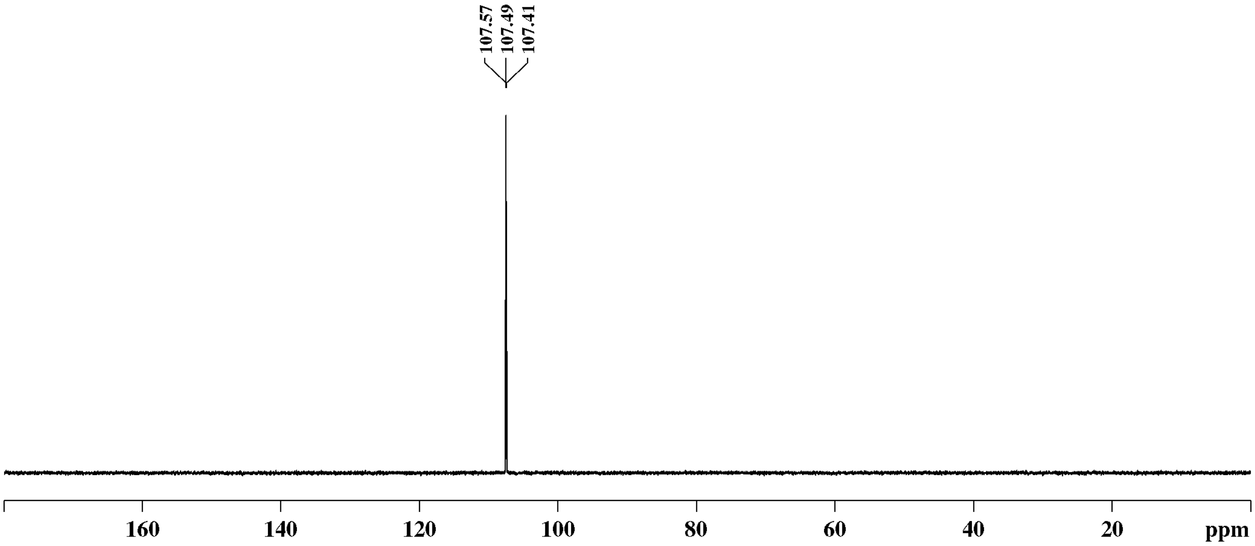

Figure S23. ^{31}P NMR spectrum of **9** in D_2O.

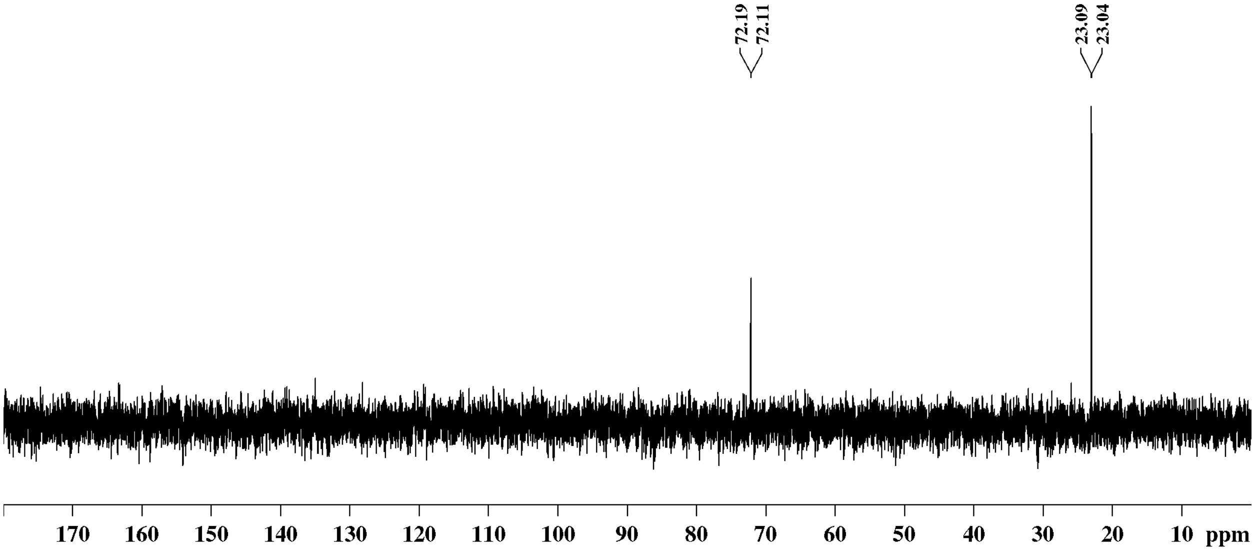

Figure S24. ^{13}C NMR spectrum of **9** in D_2O.

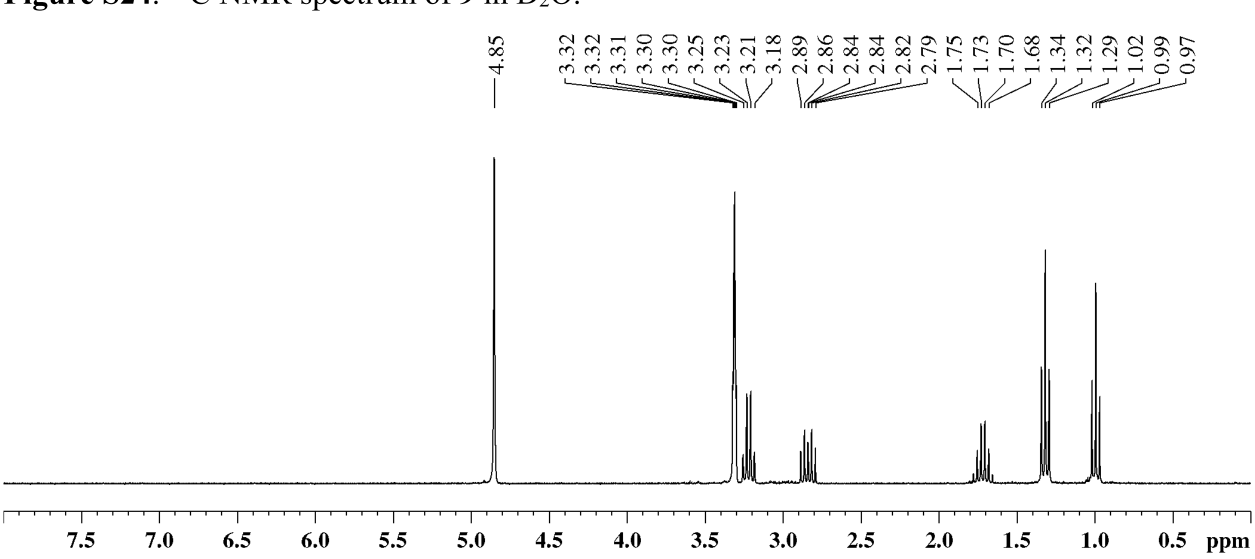

Figure S25. ^{1}H NMR spectrum of **10** in CD_3OD.

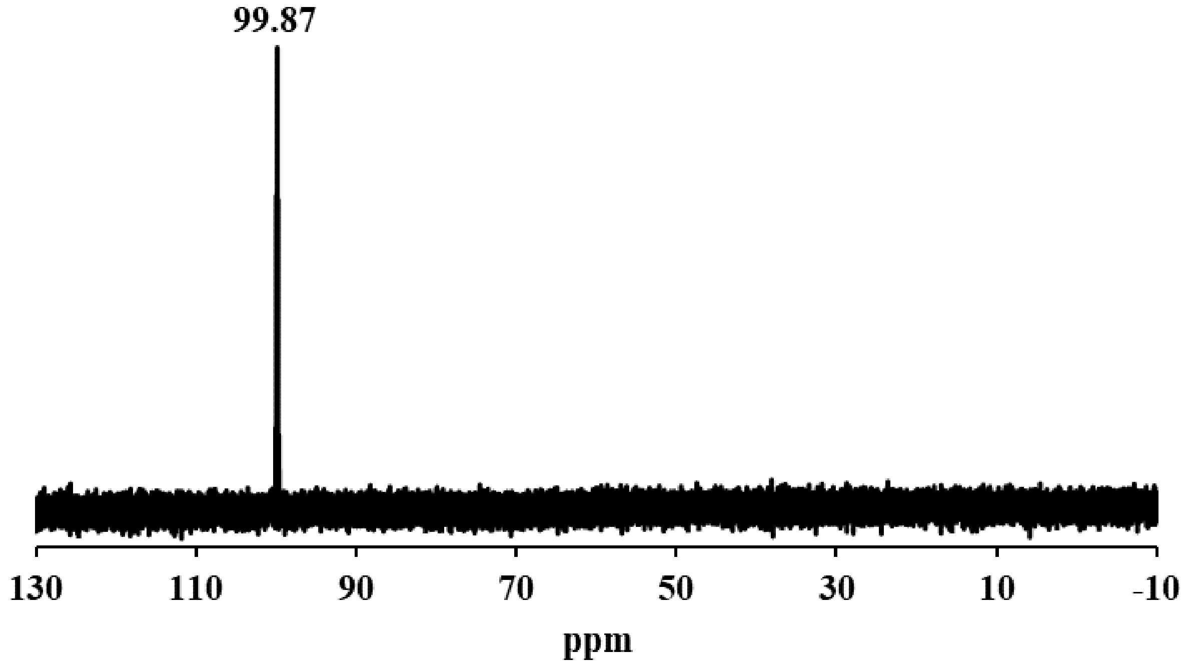

Figure S26. ^{31}P NMR spectrum of **10** in CD_3OD.

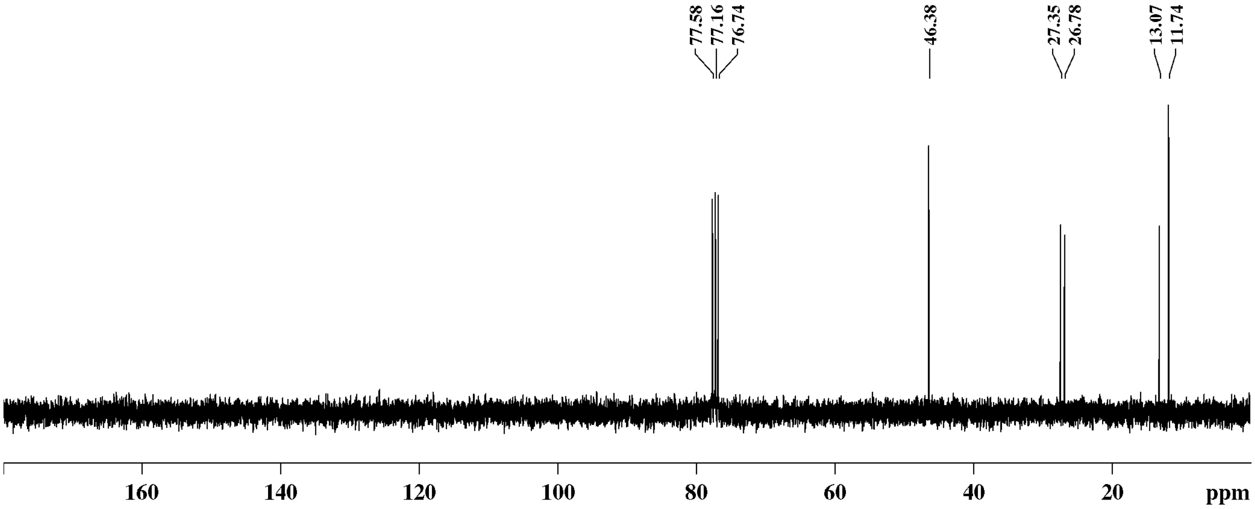

Figure S27. ^{13}C NMR spectrum of **10** in $CDCl_3$.

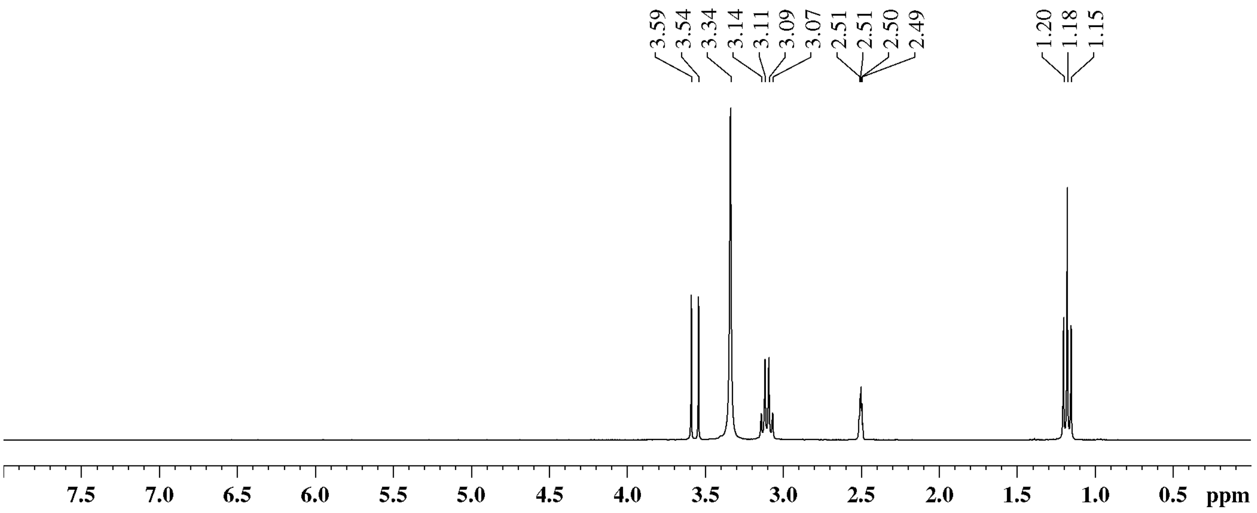

Figure S28. ^{1}H NMR spectrum of **11** in DMSO-d_6.

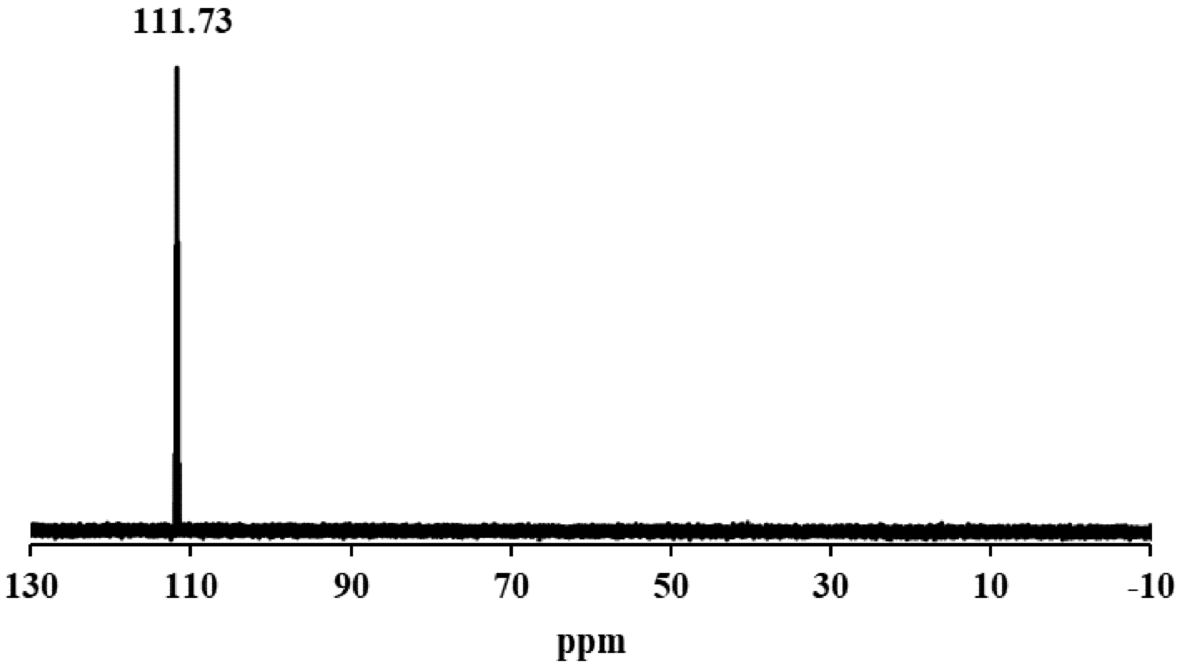

Figure S29. ^{31}P NMR spectrum of **11** in DMSO-d_6.

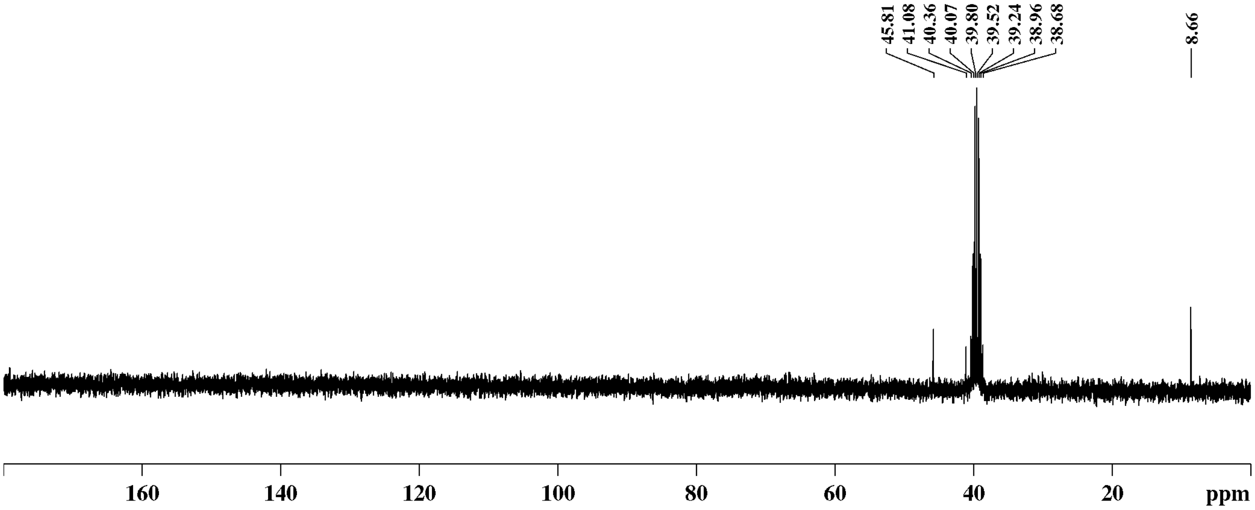

Figure S30. ^{13}C NMR spectrum of **11** in DMSO-d_6.

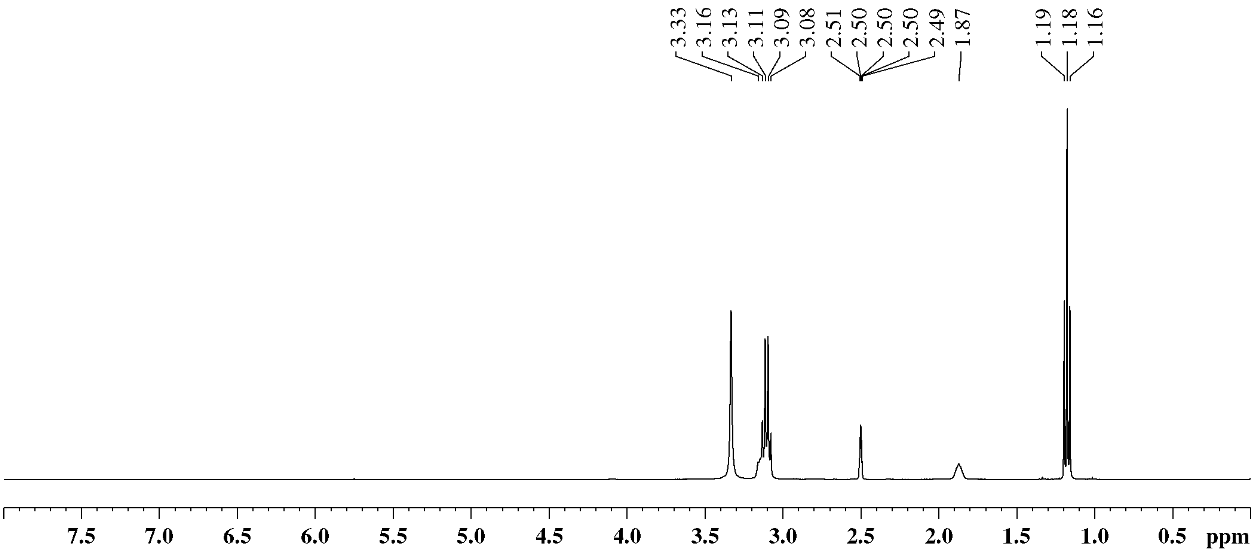

Figure S31. ^{1}H NMR spectrum of **12** in DMSO-d_6.

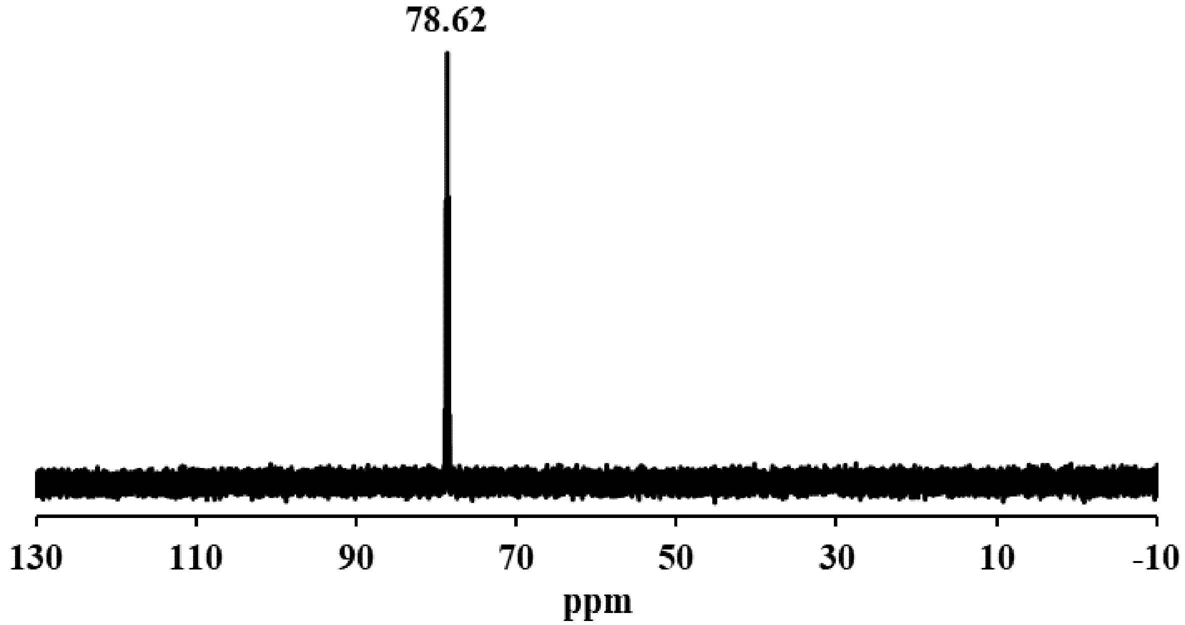

Figure S32. ^{31}P NMR spectrum of **12** in DMSO-d_6.

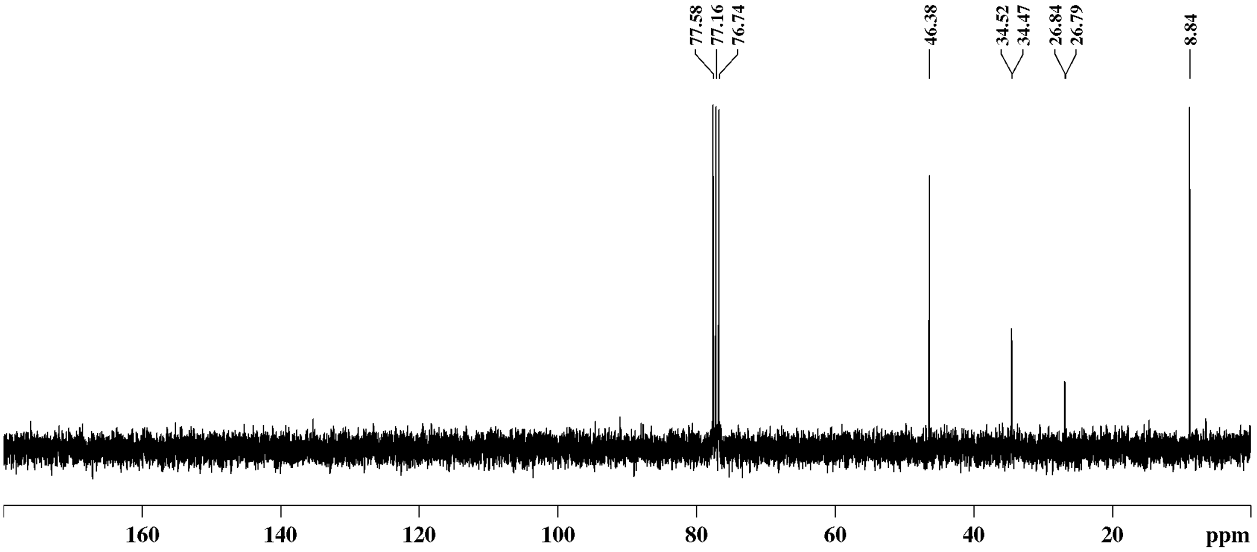

Figure S33. ^{13}C NMR spectrum of **12** in DMSO-d_6.

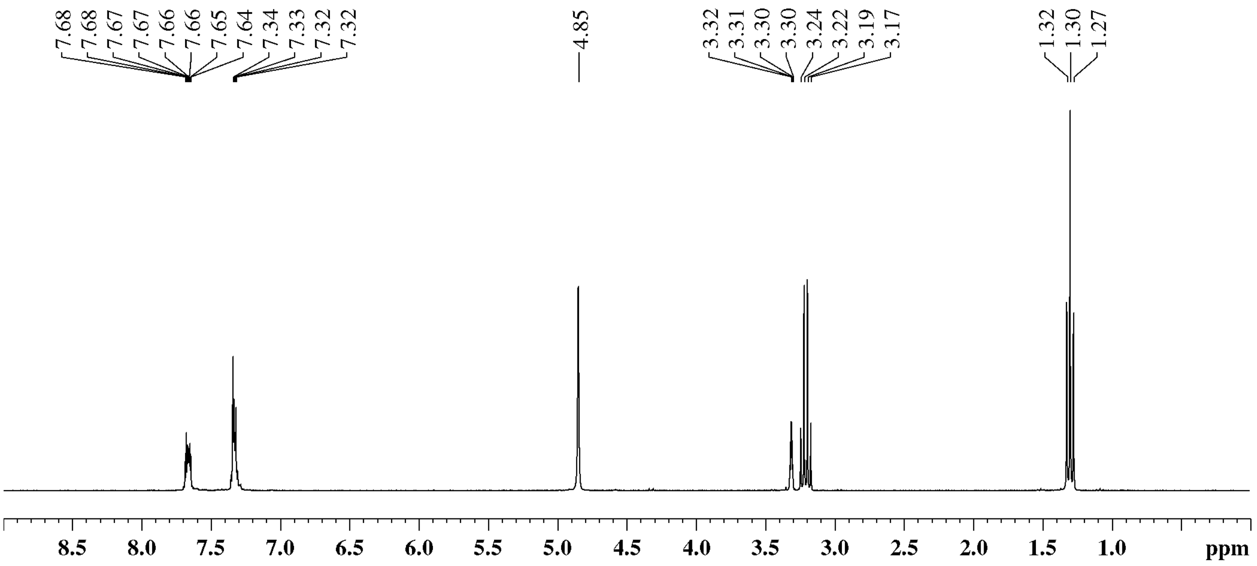

Figure S34. ^{1}H NMR spectrum of **13** in CD_3OD.

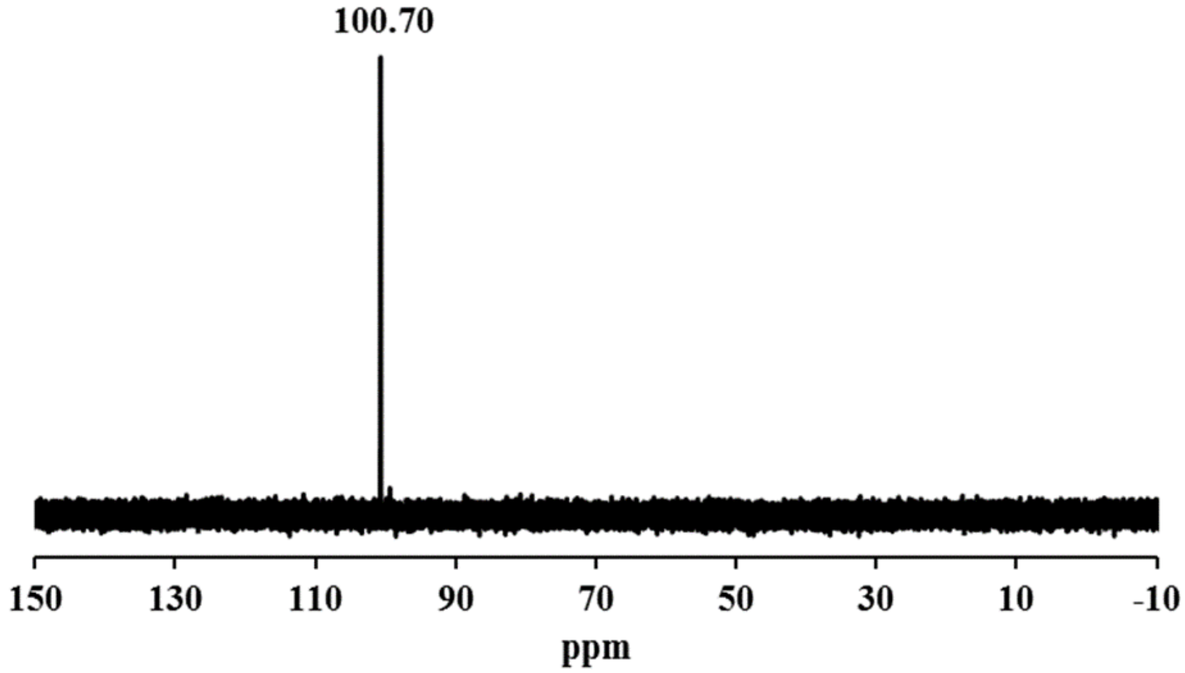

Figure S35. ^{31}P NMR spectrum of **13** in CD_3OD.

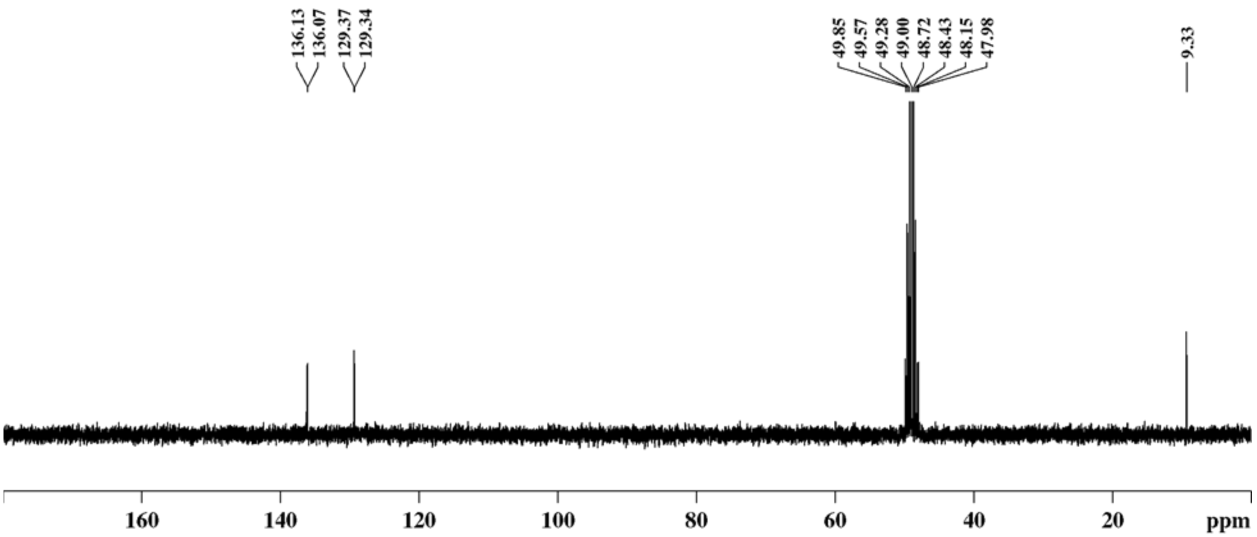

Figure S36. ^{13}C NMR spectrum of **13** in CD_3OD.

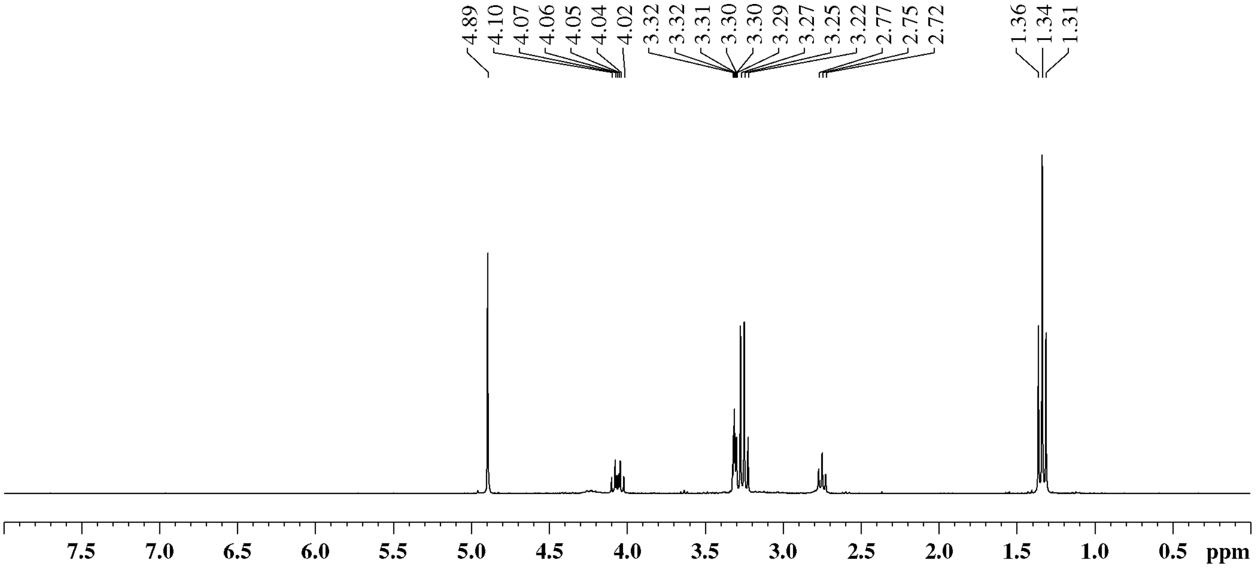

Figure S37. ^{1}H NMR spectrum of **14** in CD_3OD.

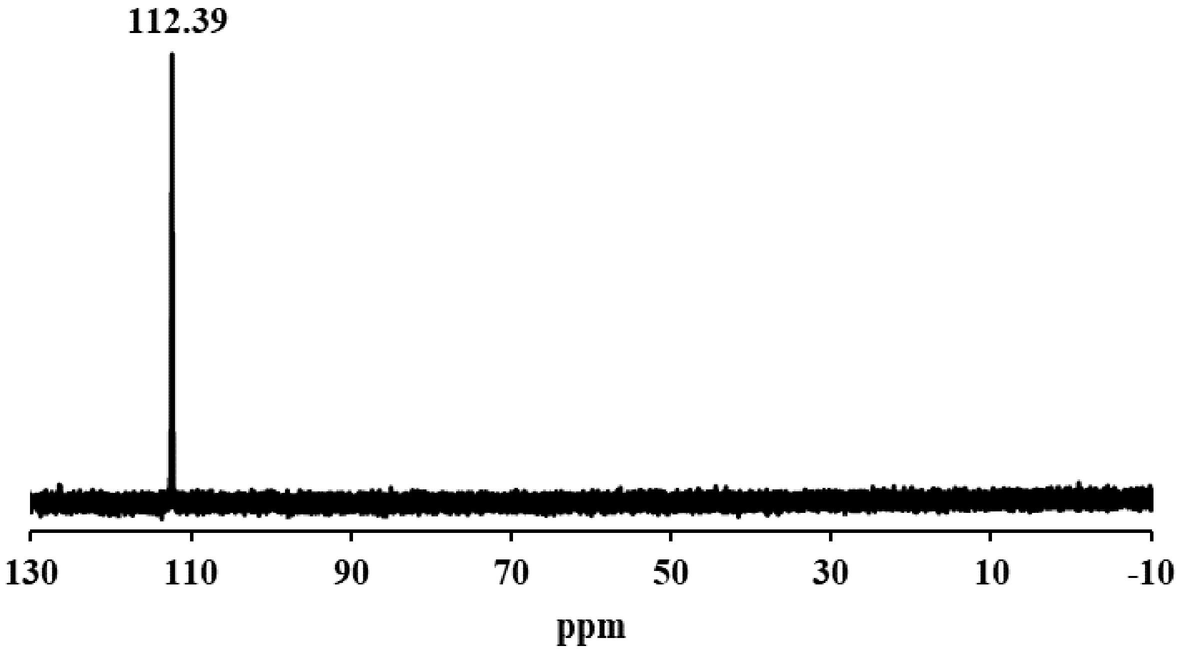

Figure S38. ^{31}P NMR spectrum of **14** in CD3OD.

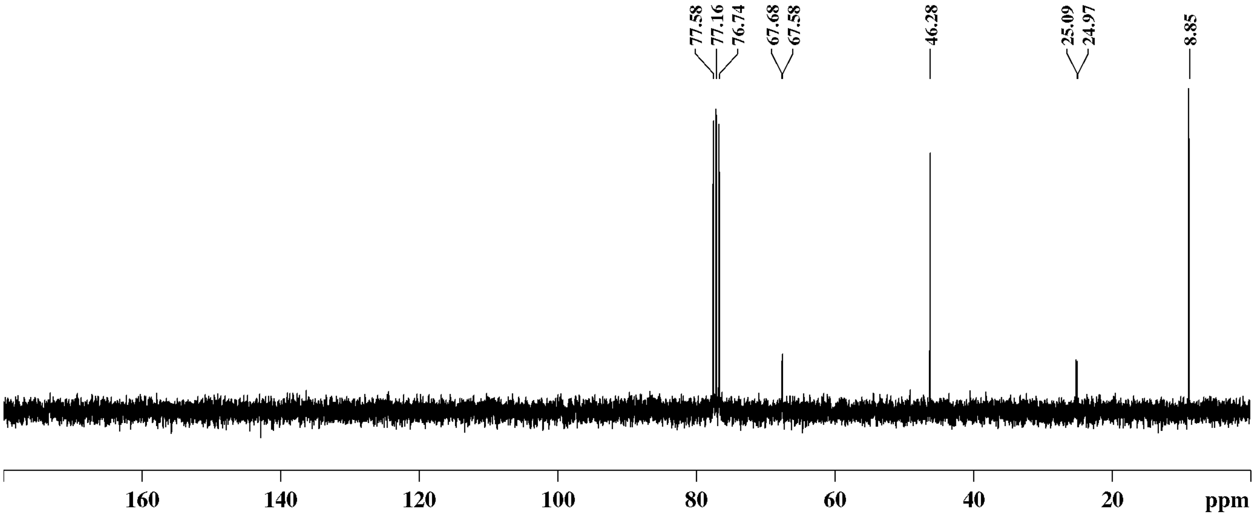

Figure S39. ^{13}C NMR spectrum of **14** in CD3OD.

Kinetic graphs of dithiophosphates used to determine the rate constants for hydrolysis.

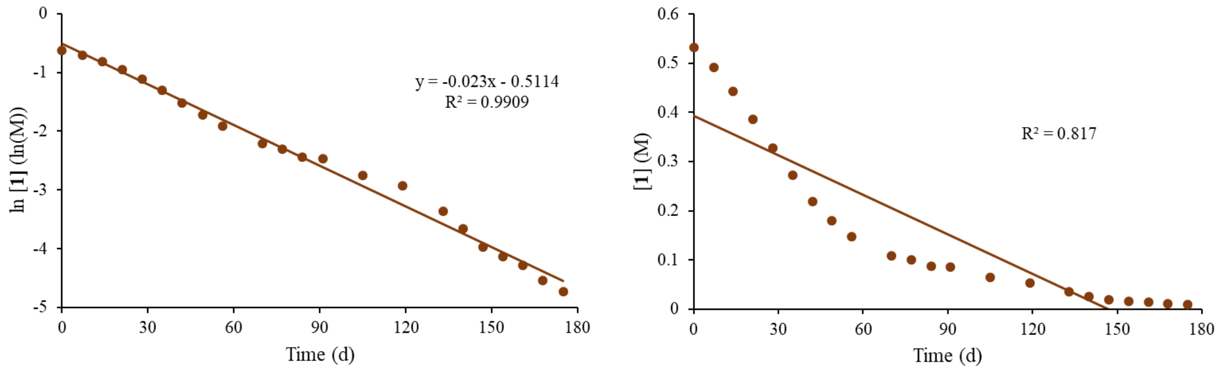

Figure S40. The kinetic graph of **1** fit to pseudo first order (left) and zero order (right) reaction kinetics showed a better fit for a psuedo first order reaction.

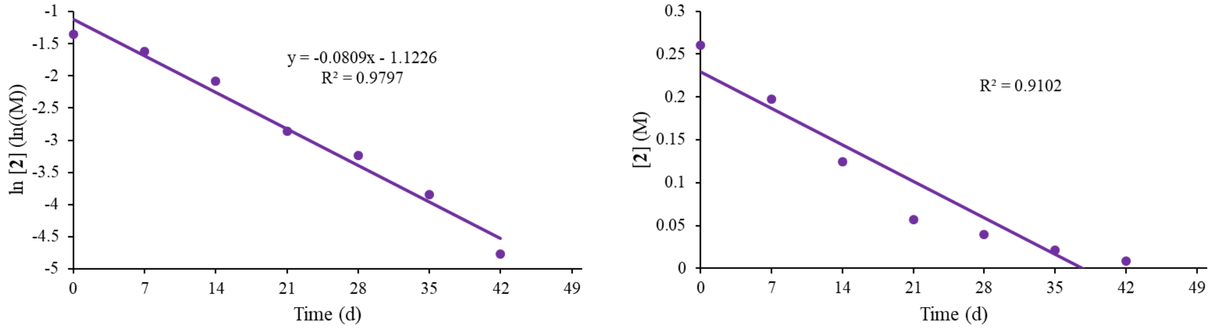

Figure S41. The kinetic graph of **2** fit to pseudo first order (left) and zero order (right) reaction kinetics showed a better fit for a psuedo first order reaction.

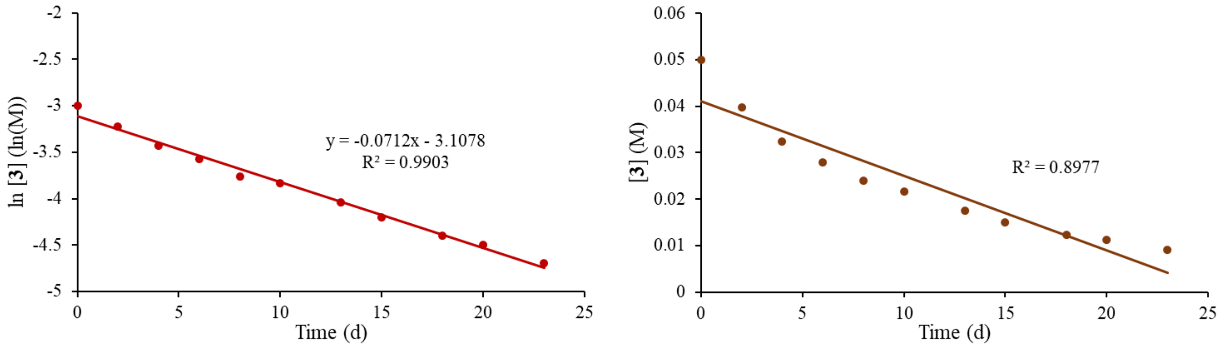

Figure S42. The kinetic graph of **3** fit to pseudo first order (left) and zero order (right) reaction kinetics showed a better fit for a psuedo first order reaction.

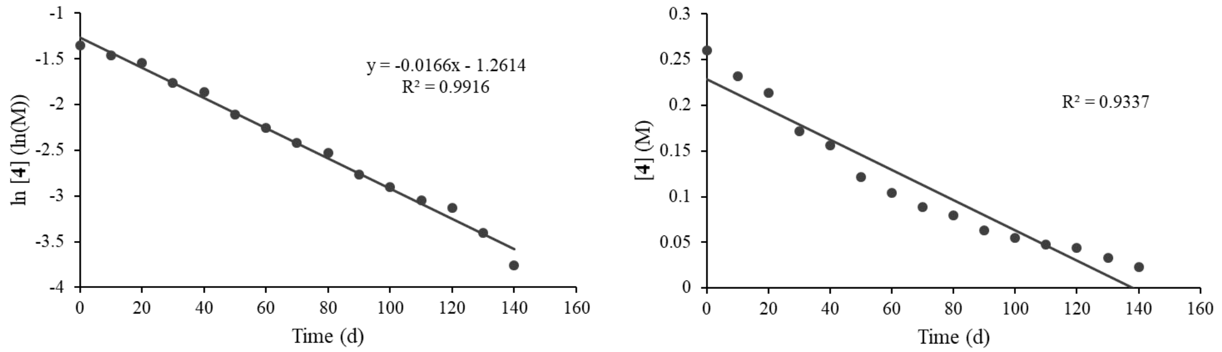

Figure S43. The kinetic graph of **4** fit to pseudo first order (left) and zero order (right) reaction kinetics showed a better fit for a psuedo first order reaction.

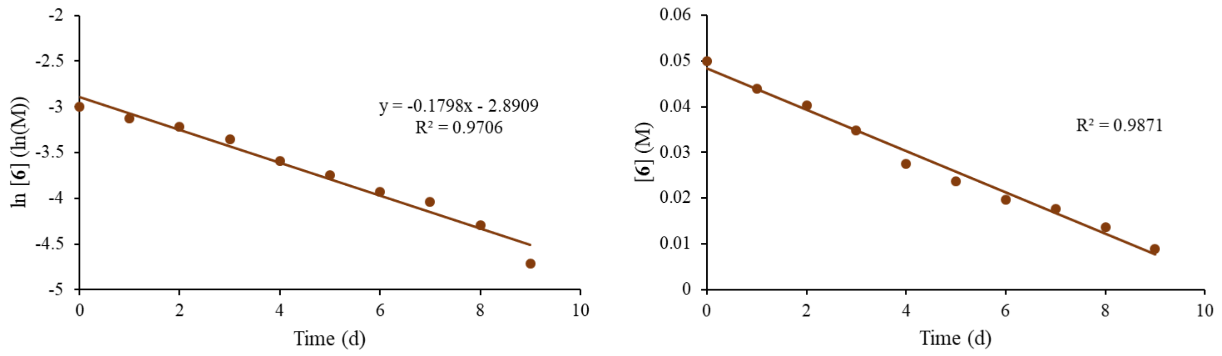

Figure S44. The kinetic graph of **6** fit to pseudo first order (left) and zero order (right) reaction kinetics showed a better fit for a psuedo first order reaction.

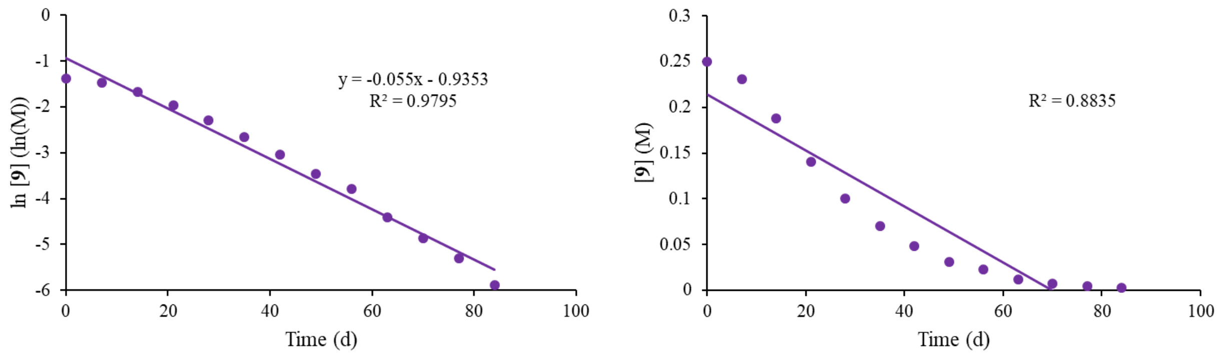

Figure S45. The kinetic graph of **9** fit to pseudo first order (left) and zero order (right) reaction kinetics showed a better fit for a psuedo first order reaction.

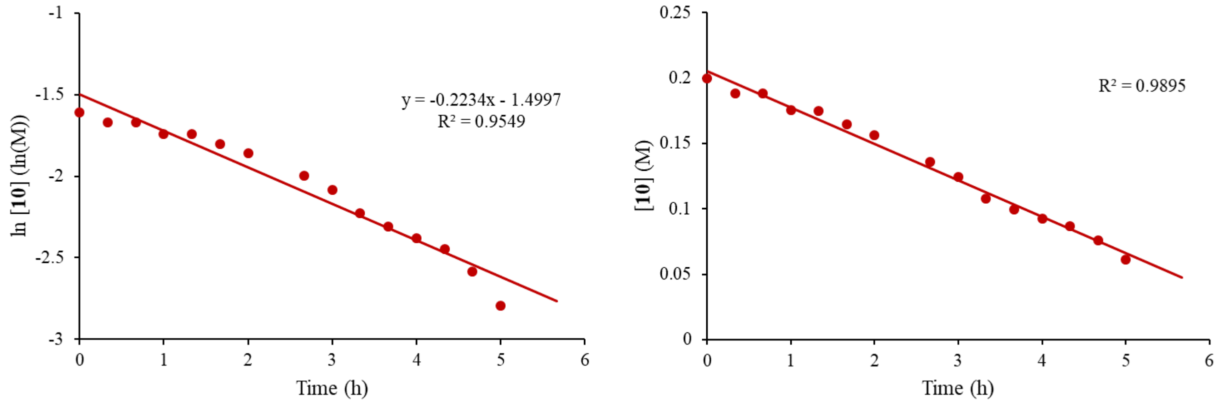

Figure S46. The kinetic graph of **10** fit to pseudo first order (left) and zero order (right) reaction kinetics showed a better fit for a psuedo first order reaction.

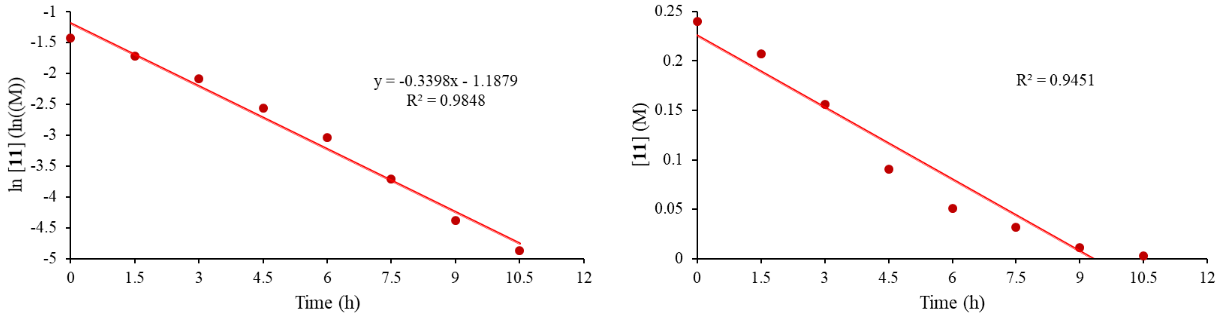

Figure S47. The kinetic graph of **11** fit to pseudo first order (left) and zero order (right) reaction kinetics showed a better fit for a psuedo first order reaction.

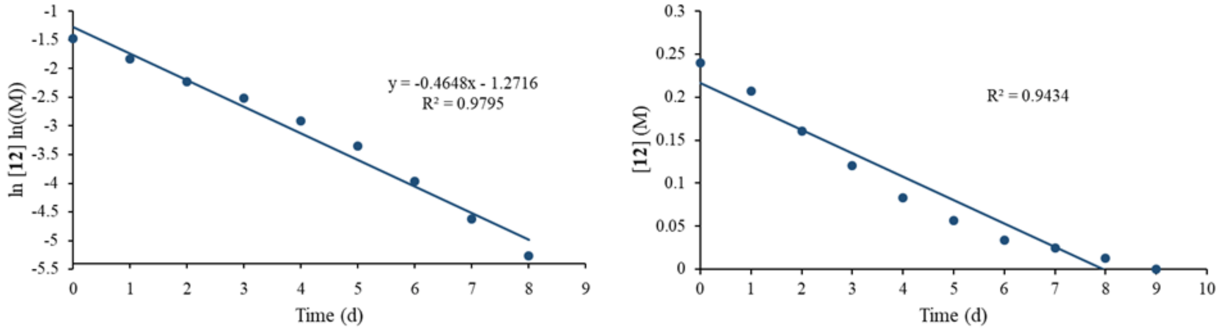

Figure S48. The kinetic graph of **12** fit to pseudo first order (left) and zero order (right) reaction kinetics showed a better fit for a psuedo first order reaction.

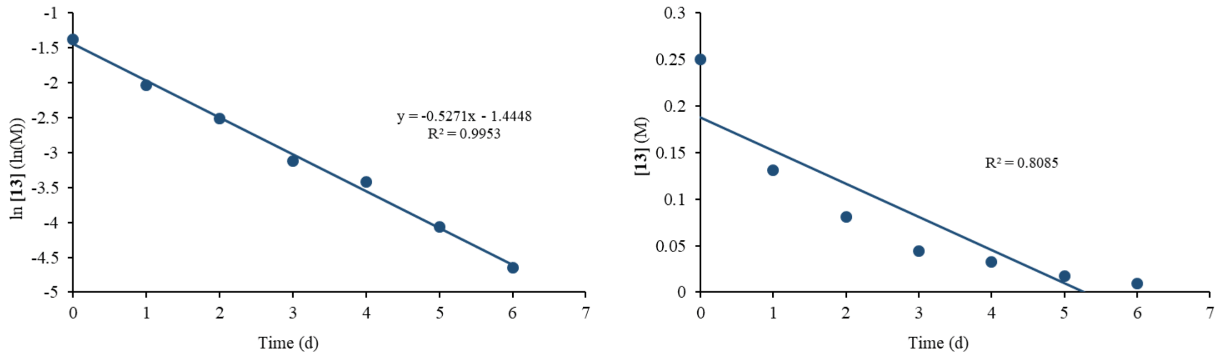

Figure S49. The kinetic graph of **13** fit to pseudo first order (left) and zero order (right) reaction kinetics showed a better fit for a psuedo first order reaction.

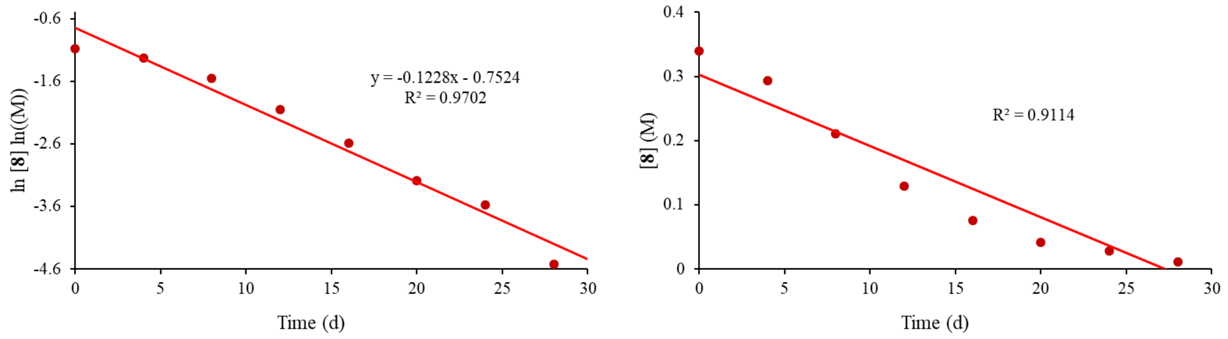

Figure S50. The kinetic graph of **8** at room temperature fit to pseudo first order (left) and zero order (right) reaction kinetics showed a better fit for a psuedo first order reaction.

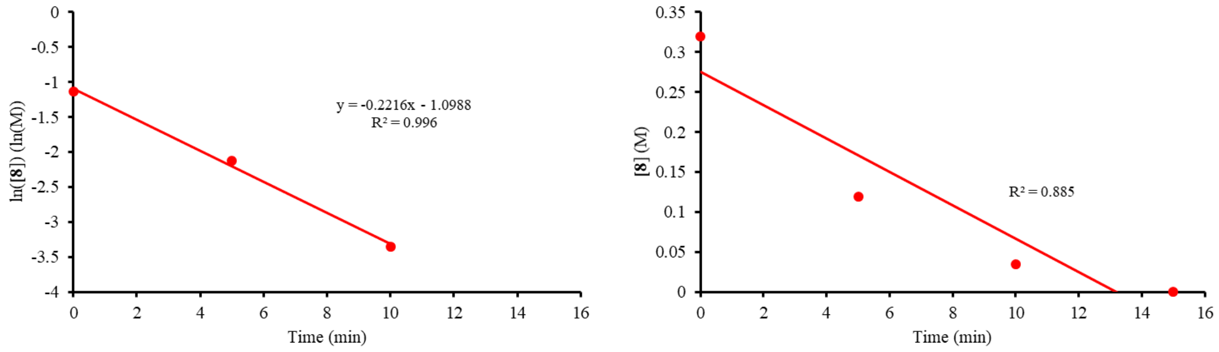

Figure S51. Kinetic graph of **8** at 85 °C fit to pseudo first order (left) and zero order (right) reaction kinetics showed a better fit for a pseudo first order reaction. The low number of data points is due to the fast degradation at 85 °C.

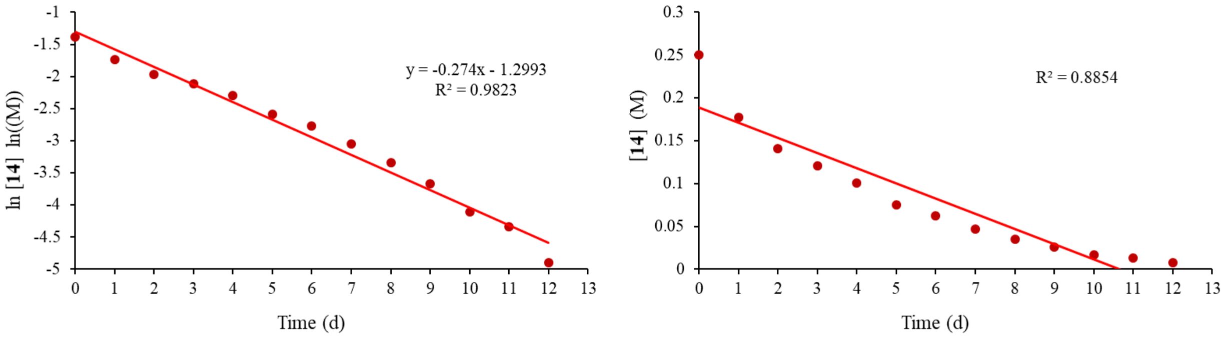

Figure S52. The kinetic graph of **14** at room temperature fit to pseudo first order (left) and zero order (right) reaction kinetics showed a better fit for a psuedo first order reaction.

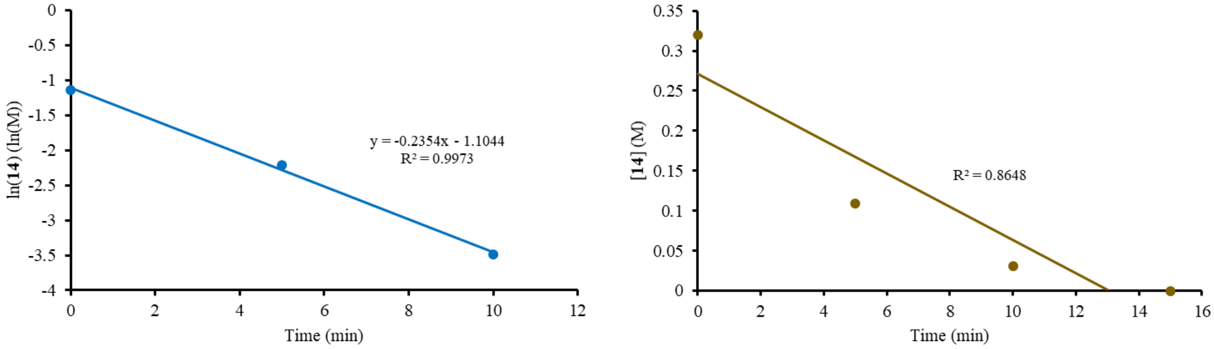

Figure S53. Kinetic graph of **14** at 85 °C fit to pseudo first order (left) and zero order (right) reaction kinetics showed a better fit for a pseudo first order reaction. The low number of data points is due to the fast degradation at 85 °C.

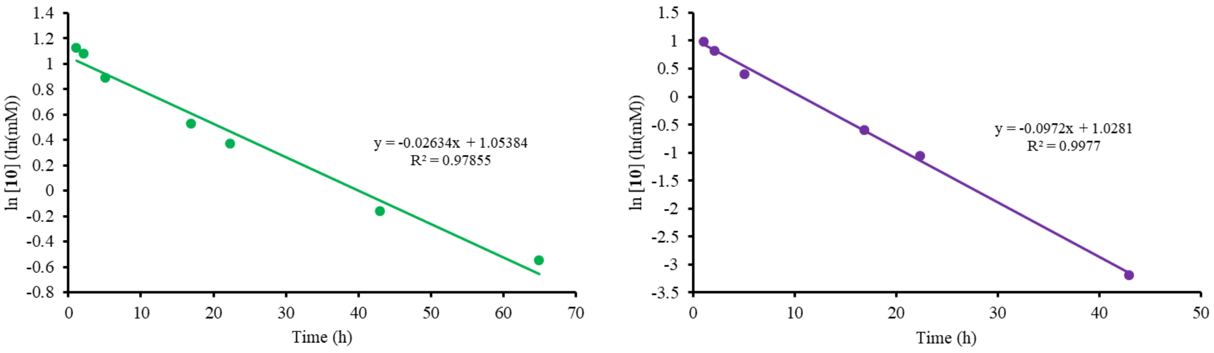

Figure S54. The graphs of the hydrolysis of **10** at 60 °C (left) and 70 °C (right) are shown.

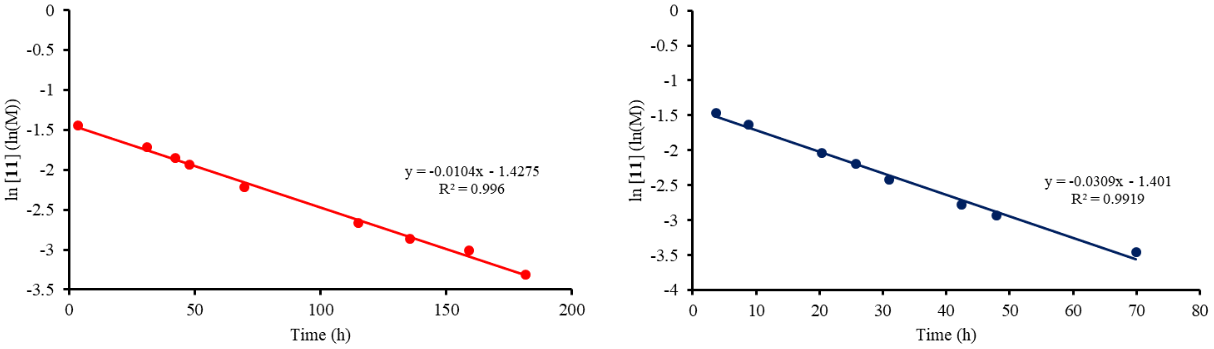

Figure S55. The graphs of the hydrolysis of **11** at 60 °C (left) and 70 °C (right) are shown.

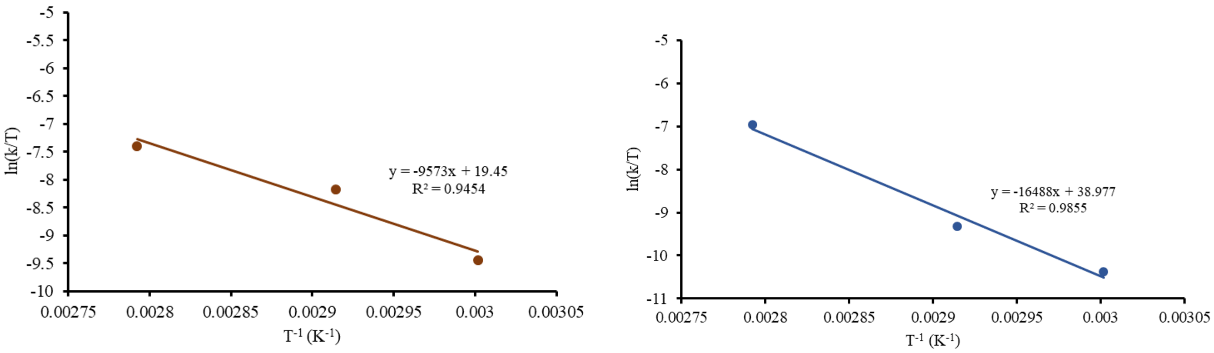

Figure S56. Eyring plots of **10** (left) and **11** (right) to obtain entropy and enthalpy values.

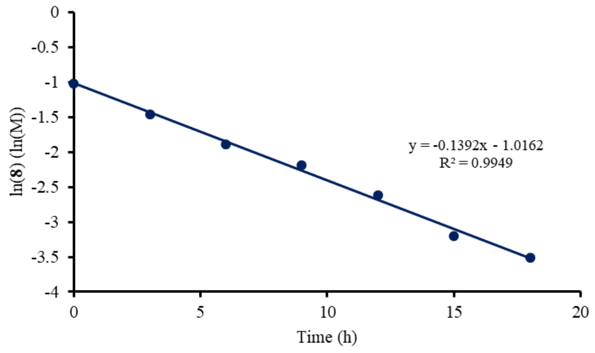

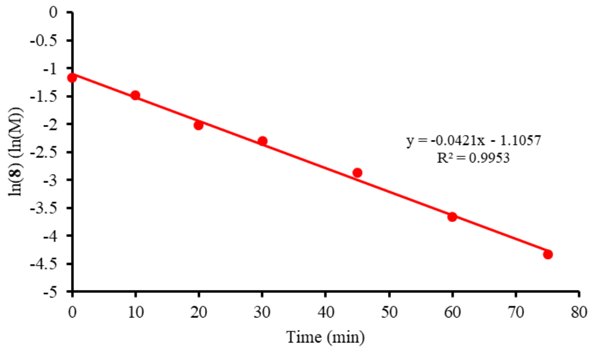

Figure S57. The graphs of hydrolysis of **8** at 45 °C (left) and 60 °C (right) are shown.

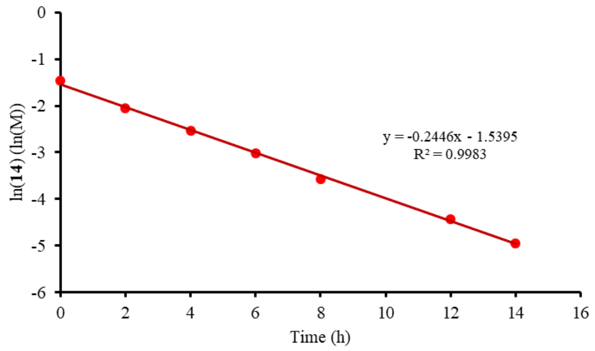

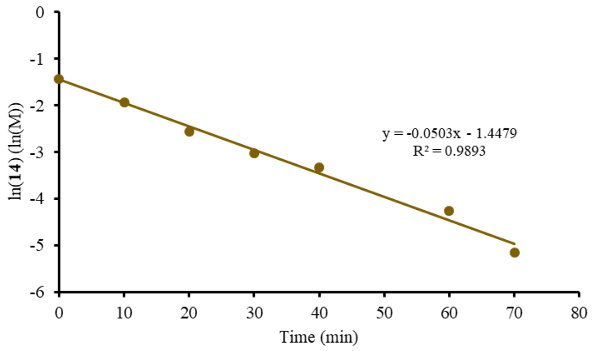

Figure S58. The graphs of hydrolysis of **14** at 45 °C (left) and 60 °C (right) are shown.

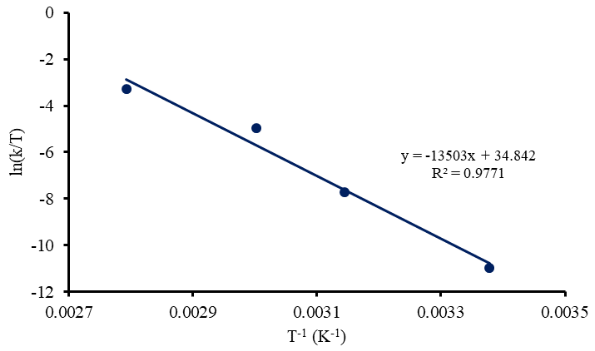

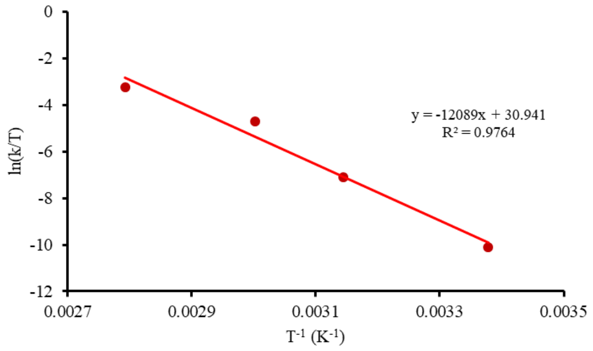

Figure S59. Eyring plots of **8** (left) and **14** (right) to obtain entropy and enthalpy values.

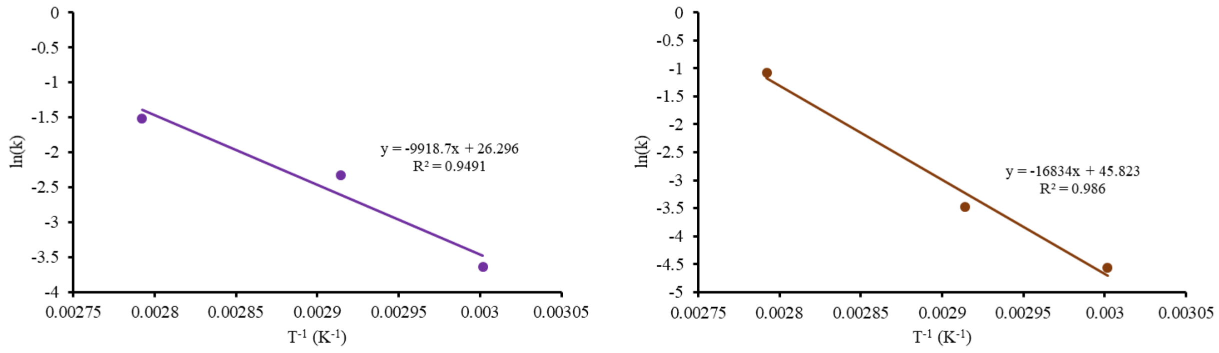

Figure S60. Arrhenius plots of **10** (left) and **11** (right) to extrapolate a rate constant at room temperature.

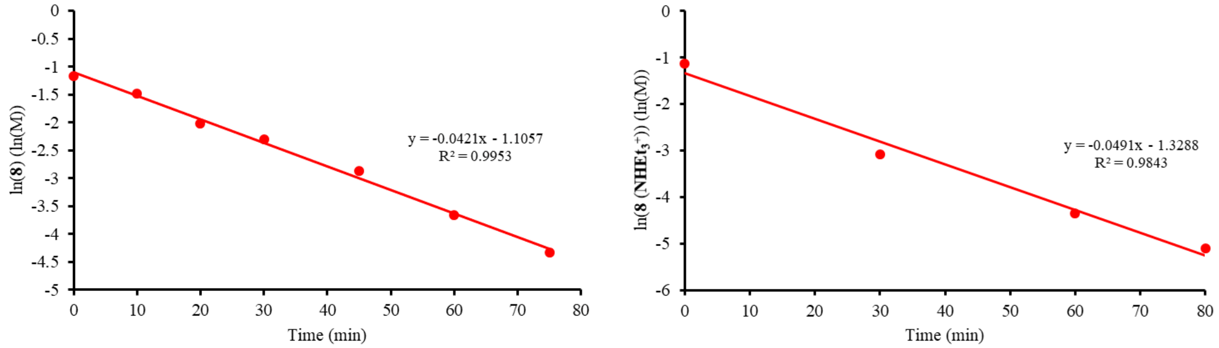

Figure S61. The graphs of hydrolysis of **8** (left) and the triethylamine salt of **8** (**8** $NHEt_3^+$) (right) at 60 °C showed a similar rate of hydrolysis.

H_2S release graphs of compound **7** and **13** that gave no measurable release of H_2S measured using an H_2S sensitive electrode.

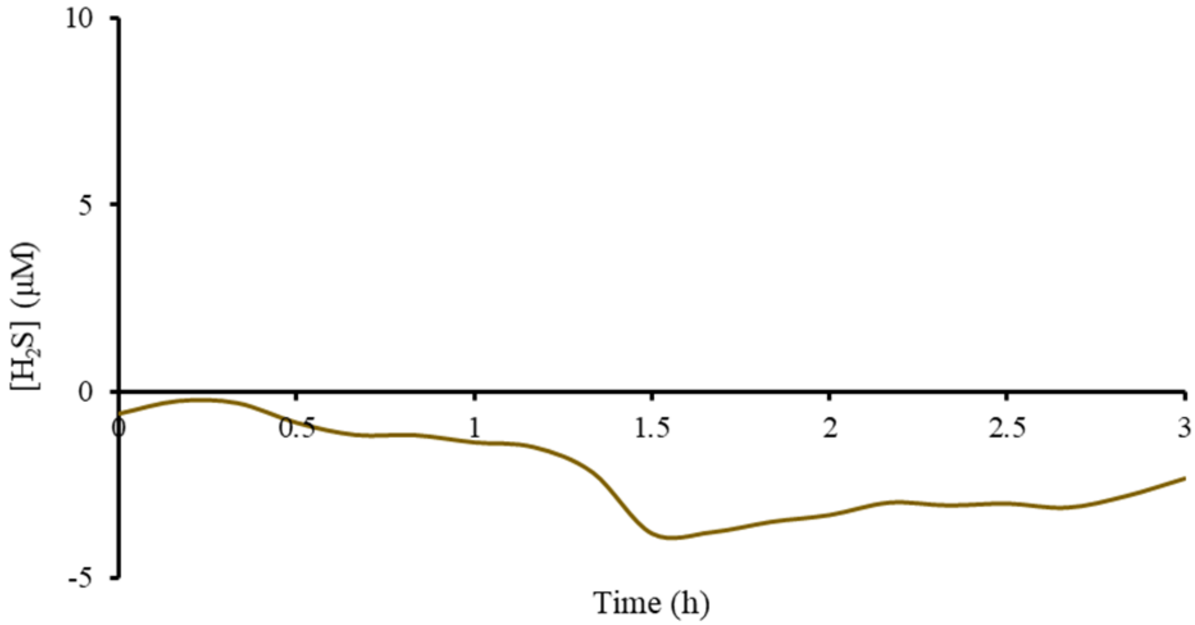

Figure S62. The concentration of H_2S from an aqueous solution of 50 mM **7** is shown. The concentration of H_2S was measured using an H_2S sensitive electrode. The concentration of H_2S in the buffer solution was measured for 0.5 h to provide a baseline of no H_2S, and then **7** was added and no increase in concentration of H_2S was observed. This chemical was insoluble and did not release a measurable amount of H_2S.

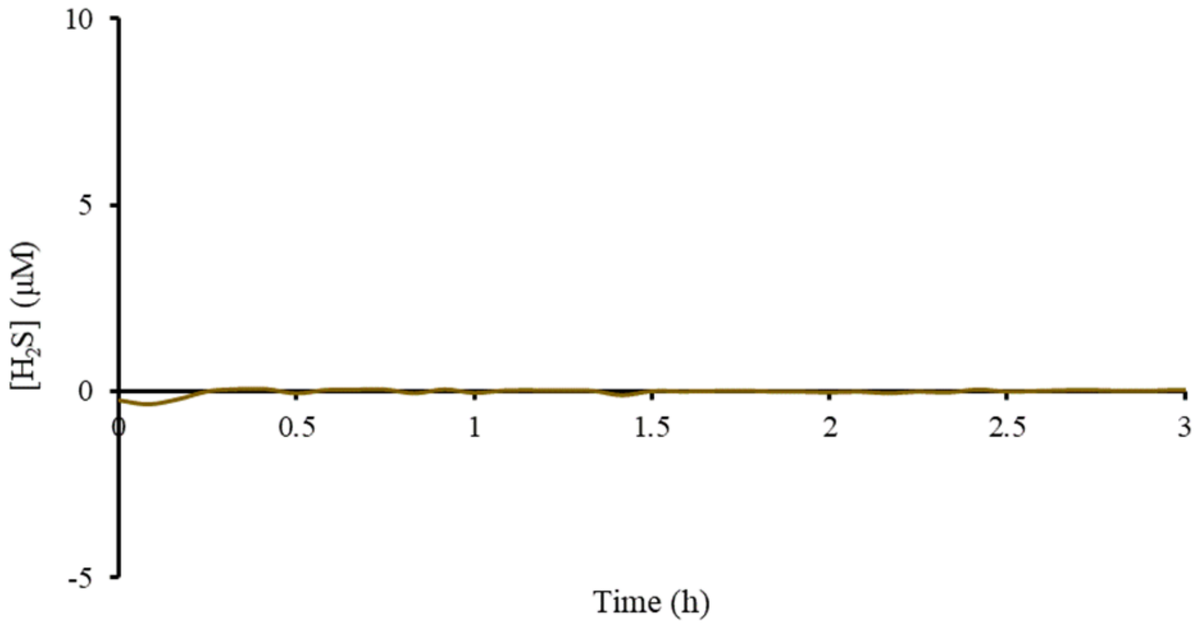

Figure S63. The concentration of H_2S from an aqueous solution of 50 mM **13** is shown. The concentration of H_2S was measured using an H_2S sensitive electrode. The concentration of H_2S in the buffer solution was measured for 0.5 h to provide a baseline of no H_2S, and then **13** was

added and no increase in concentration of H_2S was observed. This chemical was insoluble and did not release a measurable amount of H_2S.

References

1. Kumar, A.; Dinesh, J.; Kour, S.; Hundal, M. S.; Pandey, S. K., Convenient Route to Alkylene Dithiophosphato Ligands: Synthesis and Crystallographic Analysis of [OCH2CMe2CH2OPS2HNEt3]. *J. Chem. Crystallogr.* **2012**, *42*, 299-304.
2. Meisel, M.; Donath, C., Reaction of Dithiophosphoric Acid Chloride Betaine with Bifunctional Compounds - A New Route to Cyclic Dithiophosphates. *Phosphorus Sulfur Silicon Relat. Elem.* **1983**, *18*, 159-162.
3. Engel-Andreasen, J.; Wich, K.; Laursen, J. S.; Harris, P.; Olsen, C. A., Effects of Thionation and Fluorination on Cis–Trans Isomerization in Tertiary Amides: An Investigation of N-Alkylglycine (Peptoid) Rotamers. *J. Org. Chem.* **2015**, *80*, 5415-5427.
4. Nizamov, I. S.; Sofronov, A. V.; Al'metkina, L. A.; Musin, R. Z.; Cherkasov, R. A., Synthesis of optically active O,O-Di-L-(−)- and O,O-Di-D-(+)-menthyldithiophosphoric acids and their ammonium salts. *Russ. J. Gen. Chem.* **2010**, *80*, 1722-1723.
5. Radha, A.; Kumar, S.; Sharma, D.; Jassal, A. K.; Zaręba, J. K.; Franconetti, A.; Frontera, A.; Sood, P.; Pandey, S. K., Indirect influence of alkyl substituent on sigma-hole interactions: The case study of antimony(III) diphenyldithiophosphates with covalent Sb-S and non-covalent Sb···S pnictogen bonds. *Polyhedron* **2019**, *173*, 114126.
6. Nizamov, I. S.; Gabdullina, G. T.; Al'metkina, L. A.; Shamilov, R. R.; Batyeva, E. S.; Cherkasov, R. A., (1S)-endo-(−)-borneol in the synthesis of optically active phosphorus dithioacids. *Russ. J. Gen. Chem.* **2012**, *82*, 1751-1752.
7. Burn, A. J.; Dewan, S. K.; Gosney, I.; Tan, P. S. G., Phosphorus-31 nuclear magnetic resonance studey of the mechanism and kineticsof the hydrolysis of zinc(II) O,O-diethyl dithiophoshate and somerelated compounds. *J. Chem. Soc., Perkin Trans. 2* **1990**, 753-758.
8. Guo, B.; Njardarson, J. T., Z-Selective ring opening of vinyl oxetanes with dialkyl dithiophosphate nucleophiles. *Chem. Commun.* **2013**, *49*, 10802-10804.
9. Comel, A.; Kirsch, G.; Paquer, D., New Synthetic Way for the Preparation of 1,3,2-Oxathiaphospholane or 1,3,2-Oxathiaphosphorinane 2-Sulfide Derivatives. *Phosphorus Sulfur Silicon Relat. Elem.* **1994**, *89*, 25-29.
